# The impact of environmental and climatic variables on genetic diversity and plant functional traits of the endangered tuberous orchid (*Orchis mascula* L.)

**DOI:** 10.1038/s41598-022-19864-4

**Published:** 2022-11-17

**Authors:** Mohammad Mafakheri, Mehdi Bakhshipour, Mina Omrani, Hamid Gholizadeh, Najmeh Rahimi, Ali Mobaraki, Mehdi Rahimi

**Affiliations:** 1 Department of Plant Sciences, University of California - Davis, Davis, CA 95616 USA; 2 Department of Horticultural Sciences, Faculty of Agricultural Sciences, University of Guilan, P.O. Box, Rasht, 41635-1314 Iran; 3 School of Science and Technology, Faculty of Science, Agriculture, Business and Law, University of New England, Armidale, NSW 2351 Australia; 4 Department of Plant Biology, Faculty of Basic Sciences, University of Mazandaran, Babolsar, Mazandaran Iran; 5 Department of Chemistry and Biochemistry, New Mexico State University, Las Cruces, NM USA; 6 Department of Biotechnology, Institute of Science and High Technology and Environmental Sciences, Graduate University of Advanced Technology, Kerman, Iran

**Keywords:** Ecology, Genetics, Plant sciences

## Abstract

Understanding how environmental factors shape patterns of genetic and phenotypic variations in a species is necessary for conservation and plant breeding. However, these factors have not yet been completely understood in tuberous orchid species used to make ‘Salep’, an important ingredient in traditional medicine and beverages in middle eastern countries and India. In many areas, increasing demand has pushed species to the brink of extinction. In this study, 198 genotypes from 18 populations of the endangered species *Orchis mascula* L. spanning a large-scale climatic gradient in northern Iran were used to investigate patterns of genetic diversity and plant functional traits. Populations were sampled from three land cover types (woodland, shrubland, and pastureland/grassland). Plant height, stem length, number of flowers, bulb fresh and dry weight, glucomannan, and starch concentrations showed high variation among populations and were significantly related to land cover type. In general, genetic diversity was high, particularly in those from eastern Hyrcanian; additionally, populations showed a high level of genetic differentiation (*G'*_st_ = 0.35) with low gene flow (*Nm* = 0.46). The majority of genetic differentiation occurred within populations (49%) and land cover types (20%). The population structural analysis using the AFLP marker data in K = 4 showed a high geographical affinity for 198 *O. mascula* genotypes, with some genotypes having mixed ancestry. Temperature and precipitation were found to shape genetic and phenotypic variation profoundly. Significant isolation by the environment was observed, confirming the strong effect of environmental variables on phenotypic and genetic variation. Marker-trait association studies based on MLM1 and MLM2 models revealed significant associations of P-TGG + M-CTT-33 and E-AGG + M-CGT-22 markers with plant height and glucomannan content. Overall, a combination of large-scale climatic variables and land cover types significantly shaped genetic diversity and functional trait variation in *O. mascula* populations.

## Introduction

Environmental factors play a key role in shaping patterns of genetic diversity and phenotypic variation in plant species^1^. They often display plasticity and genetic variation along environmental gradients, as these gradients can affect gene flow among populations or create extreme abiotic conditions. Interactions between the environment, phenotypic and genetic diversity have been studied in different plant species at the population level, emphasizing the effect of climate gradients (e.g., temperature and precipitation)^2,3^. Genetic diversity is crucial since it can act as a buffer against abiotic or biotic stress, anthropological disturbance, inbreeding depression, and interaction with invasive species^2,4^. Populations with a history of overexploitation and fragmentation can face genetic depletion that can severely impact the survival and evolution of the species by negatively affecting fitness characteristics^5–7^. Under extreme conditions such as prolonged drought or high temperature, high genetic diversity is a significant advantage given the higher possibility of having genotypes/alleles that vary in their response to extremes^8^, enabling adaptation to environmental changes^9^. A species' capability to occupy new niches can also be affected by the loss of genetic diversity as well as damaging population growth and breeding^10–12^. A population's response to the environment (land cover, climate, etc.) can additionally be examined using functional traits, traits that are thought to have a profound influence on plant fitness^13–15^. The plasticity of these traits under various environmental conditions is a fundamental mechanism that plants rely on to react to environmental changes. The convoluted relationship between genetic diversity, functional traits, and environmental heterogeneity of the locations at the population level has been the subject of quite a few studies^16,17^ and deserves more attention.

Tuberous orchids have a rich history of traditional use and have suffered from overcollection. The dried tubers of specific wild terrestrial orchids are milled to make a flour known as 'salep'^18^, which traditionally has been an essential ingredient of confectioneries^19^. In addition to conferring aromatic and flavor to formulations, it also is a thickening and stabilizing chemical agent^20^. Salep is integral to several medicines, beverages, and ice-creams^21^. Its critical rheological properties are due to the presence of polysaccharides, especially glucomannan (GLN; 16–55%)^18^, that is, natural fibers with high water solubility. These also have specific medicinal benefits such as normalizing blood sugar, inhibiting liver irregularities, and alleviating pancreas stress^22^. In recent years the harvest of tuberous orchids has increased due to high prices fueled by international demand, mainly from Iran's neighboring countries such as India, Turkey, and Pakistan^23^. Increasing public awareness of salep's benefits and growing demand is a dangerous combination that may jeopardize the long-term viability of natural populations of these orchids.

Besides the ongoing over-exploitation of the wild population of tuberous orchids, climate change may also hasten the extinction of orchid species^24,25^. Agricultural expansion, urbanization, and silviculture have exacerbated the loss of genetic diversity in orchid populations^26,27^. Such problematic circumstances have already taken their toll on orchids in Iran, and some orchid species, including *Orchis mascula* L., have been classified as endangered^26^. Therefore, conservation strategies are needed to maintain the genetic diversity in the remaining populations and assure the species' long-term viability^28,29^. However, the absence of reliable information on the orchid's population status or the ecological consequences of severe over-collection prevents the implementation of robust conservation plans.

Although in Iran, as in other countries, legislators have enacted detailed regulations in favor of germplasm protection. Unfortunately, these regulations have not been enforced properly, and current measures are not limiting the collection or marketing of natural resources, particularly orchid tubers^23,27^. Recently, numerous small-scale regional studies on the population genetic status of tuberous orchids in the west of Iran have been reported^30–32^. However, the data required to develop conservation programs to protect genetic diversity or to develop the germplasm stocks in breeding programs can only be gained through sufficiently large-scale analyses, preferably at the landscape level^33,34^. *O. mascula* is a relatively small perennial orchid widely distributed across Europe, western Asia, and northern Africa. In Iran, this species is distributed primarily in the north (Alborz Mountains) and the west (the Zagros Mountains)^35,36^. It is one of several widely collected terrestrial orchids with numerous traditional medicinal applications. Overcollection of tubers has reduced *O. mascula* population sizes to the point that this species is now endangered; thus, fundamental large-scale studies of the functional traits and genetic diversity responses are needed. This study aimed to sample a high number of individuals from 18 populations of the terrestrial orchid *O. mascula* in northern Iran to address the following questions: (1) does phenotypic plasticity in functional traits exist in response to environmental heterogeneity? (2) how is genetic diversity and population structure affected by the land cover pattern and climatic variables? (3) do any population(s) have superior biochemical and phenotypic traits to be utilized in breeding programs?

## Results

### Phenotypic and chemotypic plasticity in response to environmental conditions

Land cover significantly affected most of the traits (*p* ≤ 0.05 and *p* ≤ 0.01); the same applies to the population effect (Table 1). The variance component of PC1 and PC2 under land cover effect was considerably high (54.88 and 34.71, respectively). As agriculturally important characters, starch content and bulb fresh weight (BFW; 154.75 and 32.34, respectively) indicated the highest variance component, while floral traits generally had the lowest values (Table 1). Plant height (PLH) with 80.97% and PC1 with 74.52% showed the highest percent of variation (%var). In the other source of variation, populations as 'random effect,' variance component was generally low, and PC1(48.77) and glucomannan (GLN, 22.71) had the highest values. The two essential traits, BFW and dry bulb weight (BDW) observed to have the highest %var (83.10 and 64.49) among the traits. Interestingly, %var among individuals within populations (error) for floral traits was often higher than other traits.

**Table 1:** Results of unbalanced REML mixed-model ANOVA of first two principal components and 19 phenotypic and 3 biochemical traits for 198 individual plants from 18 natural populations.

Traits	Source of variation							
	Land cover			Populations			Individuals	
	F-ratio	Var comp	%var	F-ratio	Var comp	%var	Var comp	%var
PCA1	7491.01**	54.88	74.52	526.89**	48.77	23.48	4.11	1.98
PCA2	341.63 ns	34.71	10.26	161.97**	14.83	74.88	2.94	14.85
Plant height (cm)	2743.99**	0.29	80.97	111.39**	10.13	14.95	2.76	4.07
Stem length (cm)	1535.42**	1.66	65.06	173.95**	15.98	29.95	2.65	4.98
Number of leaves/plant	11.59 ns	1.30	28.01	6.145**	0.55	52.79	0.20	19.18
Leaf lenght (cm)	70.96 ns	7.05	26.54	41.46**	3.79	60.54	0.80	12.90
Leaf width (cm)	74.39**	20.88	51.65	7.40**	0.63	25.25	0.58	23.08
Inflorescence length (cm)	372.11**	0.05	68.28	18.38**	1.55	15.05	1.72	16.66
Number of flowers	1724.93**	35.01	62.07	171.95**	15.62	29.49	4.46	8.42
Length of external tepals (cm)	3.09**	0.02	46.34	0.21**	2.01	12.81	0.04	40.84
Width of external tepals (cm)	0.0084**	0.00	10.31	0.001 ns	5.00	0.00	0.00	89.68
Length of internal tepals (cm)	1.17**	0.03	33.00	0.13**	3.00	14.90	0.03	52.08
Width of internal tepals (cm)	0.0055 ns	0.02	3.94	0.002**	6.00	9.70	0.00	86.35
Length lip (cm)	1.80**	0.02	16.23	0.13**	1.01	19.46	0.01	24.30
Width lip (cm)	1.15**	0.00	14.34	0.16**	0.01	18.79	0.01	24.85
Length of median lobe of the lip (cm)	1.17**	0.01	15.88	0.16**	0.01	10.78	0.01	23.33
Median lobe width of the lip (cm)	0.023*	0.00	17.19	0.005**	0.00	11.34	0.00	71.45
Length of lateral lobe (cm)	0.56*	-0.01	17.34	0.10**	0.00	12.49	0.00	30.16
Sidelobe width (cm)	0.13**	0.07	8.63	0.009**	0.00	17.56	0.00	33.79
Bulb F weight (g)	5.12 ns	32.34	0.00	9.70**	0.88	83.10	0.18	16.89
Bulb D weight (g)	2.46 ns	6.03	29.32	1.67**	0.15	64.49	0.01	6.18
Glucomannan (g/100 g)	1202.94*	0.19	43.14	247.57**	22.71	48.16	4.09	8.68
Startch (g/100 g)	302.68**	154.75	64.10	29.38**	2.67	28.43	0.70	7.46
Total Anthocyanins (mg/g)	15.97 ns	2.03	20.85	6.11**	0.55	59.66	0.18	19.48

Estimates for the percent of variation (%var) distributed between land cover, among populations within the land cover, and among individuals within populations (error) were estimated by treating all hierarchical levels as random effects.

*P < 0.05, **P < 0.01, ***P < 0.001.

**Figure 1:**
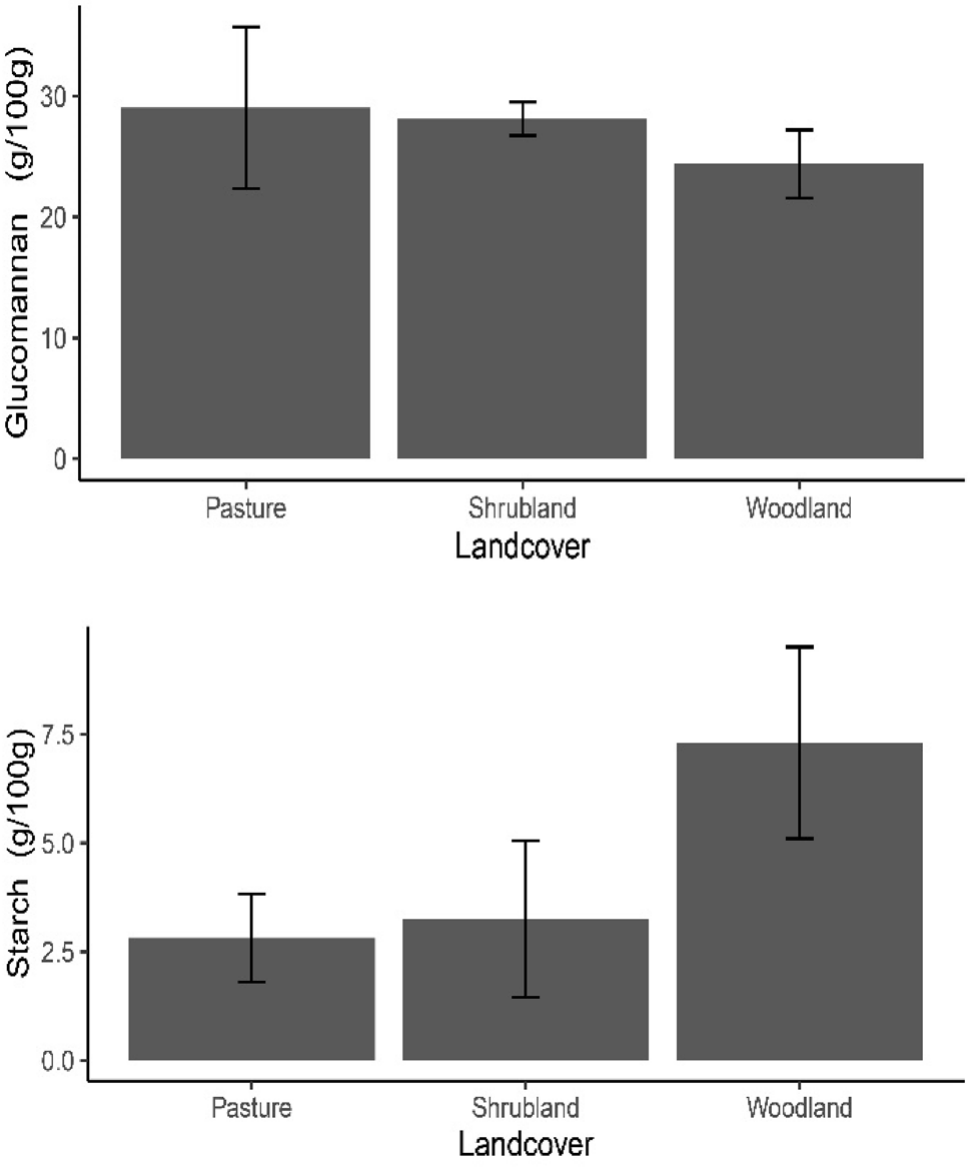
The graphs display the influence of land cover types on glucomannan (GLN; g/100 g) and starch (g/100 g) content. The difference in GLN content between pasture and woodland was significant at *P < 0.05 and nonsignificant between pasture and shrubland. Starch content under woodland land cover type versus pasture and shrubland was significant at *P < 0.05.

Phenotypic traits varied substantially between populations. For example, in traits associated with stem and leaves, the populations from the east side of the Hyrcanian ecoregion, Klaleh (KAH), Ziarat (ZIR), Azarshahr (AZS), and Maraveh Tapeh (MVT) populations overall had the highest values in general with the last population MVT, holds the most significant values. When it comes to floral traits, the variation across the populations is almost intangible (Table 2). The MVT and Talesh (TAS) populations with 3.79 and 3.27 g were found to have the highest BFW, noticeably more than the majority of the populations. The former, with 2.22 g, had the highest value for BDW. Another population from that region, KAH with 1.23 g, also showed a high value for this economically important trait. The difference between the populations became more apparent for starch and GLN contents, in which populations from Golestan, the eastern part, had higher levels, and those from Golestan were superior regarding GLN content in general. This pattern was further analyzed by assessing the relation of these two compounds against land cover, where a reverse relationship emerged (Fig. 1); the content of GLN increased in pastures/grasslands while decremented in woodland. Starch, however, seemed more affected by land cover types, but in an opposite manner, in pastureland/grassland, its content was down to 2.5 g/100 and maximized in the woodland.

**Table 2:** Means ± standard errors of 19 phenotypic and 3 biochemical traits in 198 individuals from 18 natural populations of *O. mascula*.

Pops	Stem and leaf traits						Floral traits				
	PLH	STL	NLP	LL	LW	IL	NF	LET	WET	LIT	WIT
**Guilan**											
TAS	29.86 ± 4.24	17.39 ± 4.15	3.87 ± 0.53	7.06 ± 2.02	4.28 ± 0.93	11.24 ± 1.72	16.46 ± 4.96	1.33 ± 0.48	0.28 ± 0.09	1.08 ± 0.52	0.25 ± 0.11
KHAL	25.42 ± 4.13	12.92 ± 2.21	3.27 ± 0.22	5.42 ± 2.16	4.17 ± 1.07	9.6 ± 1.79	17.24 ± 5.23	1.33 ± 0.19	0.26 ± 0.12	1.1 ± 0.58	0.25 ± 0.13
MSL	28.59 ± 4	17.23 ± 5.15	3.84 ± 0.38	6.15 ± 1.95	6.54 ± 0.86	11.51 ± 1.59	26.22 ± 5.37	1.39 ± 0.37	0.26 ± 0.13	1.06 ± 0.56	0.24 ± 0.14
DRS	27.17 ± 3.64	15.53 ± 4.44	3.49 ± 0.33	6.22 ± 2.11	4.37 ± 1.01	11.27 ± 1.57	22.57 ± 5.43	1.22 ± 0.38	0.28 ± 0.21	1.08 ± 0.63	0.25 ± 0.14
CHP	27.64 ± 5	17.62 ± 4.22	3.36 ± 0.6	5.79 ± 2.19	4.25 ± 1.1	10.64 ± 1.66	23.84 ± 4.97	1.49 ± 0.3	0.26 ± 0.12	1.24 ± 0.72	0.26 ± 0.12
KHD	30.63 ± 4.32	20.11 ± 5.58	3.54 ± 0.34	6.85 ± 1.98	5.49 ± 0.89	13.4 ± 1.71	25.33 ± 5.6	1.28 ± 0.25	0.27 ± 0.13	1.08 ± 0.74	0.23 ± 0.15
GRD	34.25 ± 4.56	23.8 ± 4.39	4.37 ± 0.36	8.63 ± 2.39	5.51 ± 1.29	12.56 ± 1.48	25.37 ± 5.21	1.42 ± 0.28	0.29 ± 0.11	1.27 ± 0.61	0.28 ± 0.13
**Mazandaran**											
JAD	24.67 ± 3.57	14.69 ± 2.08	3.18 ± 0.35	6.37 ± 1.91	2.54 ± 0.82	7.96 ± 1.45	18.35 ± 5.33	1.18 ± 0.13	0.27 ± 0.23	1.08 ± 0.51	0.26 ± 0.12
PAJ	21.63 ± 3.3	9.79 ± 5.25	3.27 ± 0.39	6.35 ± 2.07	2.34 ± 0.97	8.39 ± 1.68	13.97 ± 5.29	0.8 ± 0.14	0.27 ± 0.11	0.95 ± 0.59	0.23 ± 0.16
KJR	20.34 ± 4.02	10.87 ± 7.14	3.2 ± 0.31	6.35 ± 2.3	3.46 ± 1.2	7.92 ± 1.3	12.33 ± 5.6	0.82 ± 0.18	0.26 ± 0.1	0.82 ± 0.51	0.23 ± 0.13
KCH	19.37 ± 3.7	12.53 ± 4.51	3.23 ± 0.25	6.4 ± 1.94	3.29 ± 0.84	7.37 ± 1.33	12.02 ± 4.63	0.84 ± 0.11	0.26 ± 0.11	0.72 ± 0.51	0.23 ± 0.12
MKH	20.35 ± 3.76	9.9 ± 5.05	3.26 ± 0.19	6.32 ± 2.42	3.34 ± 1.32	8.31 ± 1.25	**10.18 ± 5.19**	0.78 ± 0.12	0.26 ± 0.1	0.73 ± 0.48	0.21 ± 0.13
**Golestan**											
PBD	30.42 ± 3.92	19.98 ± 3.54	3.78 ± 0.38	7.36 ± 2.48	5.34 ± 1.38	13.59 ± 2.08	23.67 ± 5.32	1.23 ± 0.31	0.28 ± 0.1	1.06 ± 0.54	0.25 ± 0.15
BAB	32.2 ± 4.57	19.55 ± 5.25	3.76 ± 0.37	7.35 ± 2.49	5.62 ± 1.39	13.78 ± 1.81	23.33 ± 5.33	1.38 ± 0.39	0.28 ± 0.11	1.13 ± 0.57	0.26 ± 0.13
KAH	35.4 ± 4.55	25.91 ± 4.89	4.53 ± 0.59	8.8 ± 2.37	5.64 ± 1.27	13.51 ± 1.82	27.01 ± 5.12	1.47 ± 0.16	0.3 ± 0.19	1.27 ± 0.55	0.23 ± 0.14
ZIR	**39.02 ± 4.79**	19.28 ± 3.56	3.13 ± 0.26	7.38 ± 2.5	3.75 ± 1.41	10.31 ± 1.23	22.74 ± 4.85	1.17 ± 0.25	0.27 ± 0.12	1.11 ± 0.56	0.25 ± 0.14
AZS	**39.08 ± 4.74**	21.19 ± 4.99	3.85 ± 0.33	6.22 ± 2.27	5.59 ± 1.18	14.87 ± 1.5	22.27 ± 5.61	1.18 ± 0.59	0.3 ± 0.16	1.04 ± 0.6	0.25 ± 0.12
MVT	**40.63 ± 4.31**	30.39 ± 7.71	6.05 ± 0.7	13.12 ± 2.36	6.4 ± 1.26	14.97 ± 1.6	**31.1 ± 5.13**	1.53 ± 0.37	0.3 ± 0.18	1.28 ± 0.53	0.27 ± 0.13

Pops	Floral traits						Bulbs		Biochemical traits		
	LLI	WL	LMLL	LMLW	LLL	SW	BFW	BDW	GLN	Starch	TAC
**Guilan**											
TAS	1.18 ± 0.2	0.93 ± 0.65	1.11 ± 0.61	0.36 ± 0.12	0.81 ± 0.48	0.32 ± 0.13	**3.27 ± 1.15**	0.81 ± 0.47	23.7 ± 7.65	2.9 ± 4.07	2.52 ± 1.5
KHAL	1.18 ± 0.29	0.92 ± 0.43	1.14 ± 0.49	0.37 ± 0.16	0.81 ± 0.49	0.31 ± 0.16	2.61 ± 1.14	0.47 ± 0.48	27.62 ± 6.65	2.23 ± 3.72	2.38 ± 1.17
MSL	1.16 ± 0.28	0.89 ± 0.69	1.05 ± 0.74	0.34 ± 0.14	0.82 ± 0.5	0.33 ± 0.13	1.13 ± 0.69	0.86 ± 0.58	29.29 ± 7.38	5.55 ± 3.8	2.59 ± 1.17
DRS	1.2 ± 0.29	0.85 ± 0.55	0.97 ± 0.88	0.36 ± 0.13	0.8 ± 0.54	0.33 ± 0.14	1.13 ± 0.69	0.97 ± 0.47	30.14 ± 6.49	7.53 ± 3.24	2.87 ± 1.2
CHP	1.02 ± 0.24	0.86 ± 0.63	1.05 ± 0.76	0.37 ± 0.13	0.84 ± 0.47	0.31 ± 0.11	1.1 ± 0.7	0.94 ± 0.48	30.25 ± 6.11	7.55 ± 3.49	2.85 ± 1.15
KHD	1.23 ± 0.21	0.86 ± 0.65	1.04 ± 0.74	0.38 ± 0.12	0.83 ± 0.47	0.31 ± 0.14	2.38 ± 0.94	1.1 ± 0.57	29.47 ± 6.45	7.32 ± 3.57	2.59 ± 1.08
GRD	1.37 ± 0.25	1.13 ± 0.61	1.33 ± 0.69	0.41 ± 0.12	1.04 ± 0.49	0.37 ± 0.14	2.63 ± 0.75	1.11 ± 0.52	34.49 ± 7.01	5.41 ± 3.55	1.49 ± 0.93
**Mazandaran**											
JAD	1.14 ± 0.21	0.93 ± 0.84	1.17 ± 0.32	0.38 ± 0.11	0.86 ± 0.49	0.32 ± 0.16	1.09 ± 0.64	0.94 ± 0.44	24.43 ± 6.43	8.52 ± 3.69	2.84 ± 1.21
PAJ	0.83 ± 0.24	0.61 ± 0.62	0.83 ± 0.71	0.34 ± 0.11	0.63 ± 0.44	0.22 ± 0.13	1.46 ± 0.75	0.65 ± 0.5	21.67 ± 5.55	**10.57 ± 4.07**	3.73 ± 1.03
KJR KCH	0.78 ± 0.23	0.66 ± 0.22	0.78 ± 0.7	0.34 ± 0.12	0.63 ± 0.45	0.22 ± 0.13	1.42 ± 0.69	1.16 ± 0.48	21.21 ± 6.08	10.51 ± 3.82	3.67 ± 1.07
	0.81 ± 0.18	0.63 ± 0.57	0.84 ± 0.68	0.33 ± 0.09	0.67 ± 0.44	0.22 ± 0.12	**1.46 ± 0.75**	1.2 ± 0.52	19.8 ± 6.32	**10.78 ± 4.35**	3.79 ± 1.08
MKH	0.74 ± 0.2	0.63 ± 0.39	0.83 ± 0.69	0.34 ± 0.11	0.67 ± 0.51	0.22 ± 0.12	1.51 ± 0.79	1.24 ± 0.56	19.08 ± 6.15	**11.18 ± 4.22**	3.88 ± 1.18
**Golestan**											
PBD	1.19 ± 0.19	0.94 ± 0.63	1.15 ± 0.67	0.38 ± 0.12	0.86 ± 0.45	0.33 ± 0.13	1.17 ± 0.68	1.01 ± 0.47	**31.99 ± 6.15**	6.48 ± 3.29	2.79 ± 1.01
BAB	1.16 ± 0.21	0.88 ± 0.52	1.15 ± 0.66	0.38 ± 0.1	0.82 ± 0.44	0.32 ± 0.12	1.18 ± 0.66	1.02 ± 0.45	31.29 ± 7.78	6.33 ± 3.9	2.66 ± 1
KAH	1.32 ± 0.23	1.15 ± 0.32	1.33 ± 0.7	0.42 ± 0.11	1.02 ± 0.5	0.35 ± 0.14	**1.5 ± 0.71**	1.23 ± 0.49	**34.77 ± 6.92**	5.59 ± 3.66	1.44 ± 0.89
ZIR	1.14 ± 0.22	0.89 ± 0.64	1.11 ± 0.7	0.37 ± 0.12	0.86 ± 0.45	0.31 ± 0.14	0.74 ± 0.66	1.07 ± 0.45	31.33 ± 6.95	7.21 ± 3.52	2.54 ± 0.95
AZS	1.18 ± 0.21	0.94 ± 0.62	1.12 ± 0.68	0.37 ± 0.13	0.82 ± 0.46	0.33 ± 0.14	**2.2 ± 1.74**	1.12 ± 0.63	29.23 ± 7.23	7.51 ± 3.9	2.59 ± 1.02
MVT	1.32 ± 0.21	1.17 ± 0.61	1.34 ± 0.83	0.42 ± 0.11	1.04 ± 0.52	0.37 ± 0.13	**3.79 ± 1.32**	**2.22 ± 0.47**	**38.15 ± 7.47**	4.86 ± 3.68	4.04 ± 0.96

Significant values are in bold.

### Hierarchical clustering on principal components (HCPC) and cluster analysis

**Figure 2:**
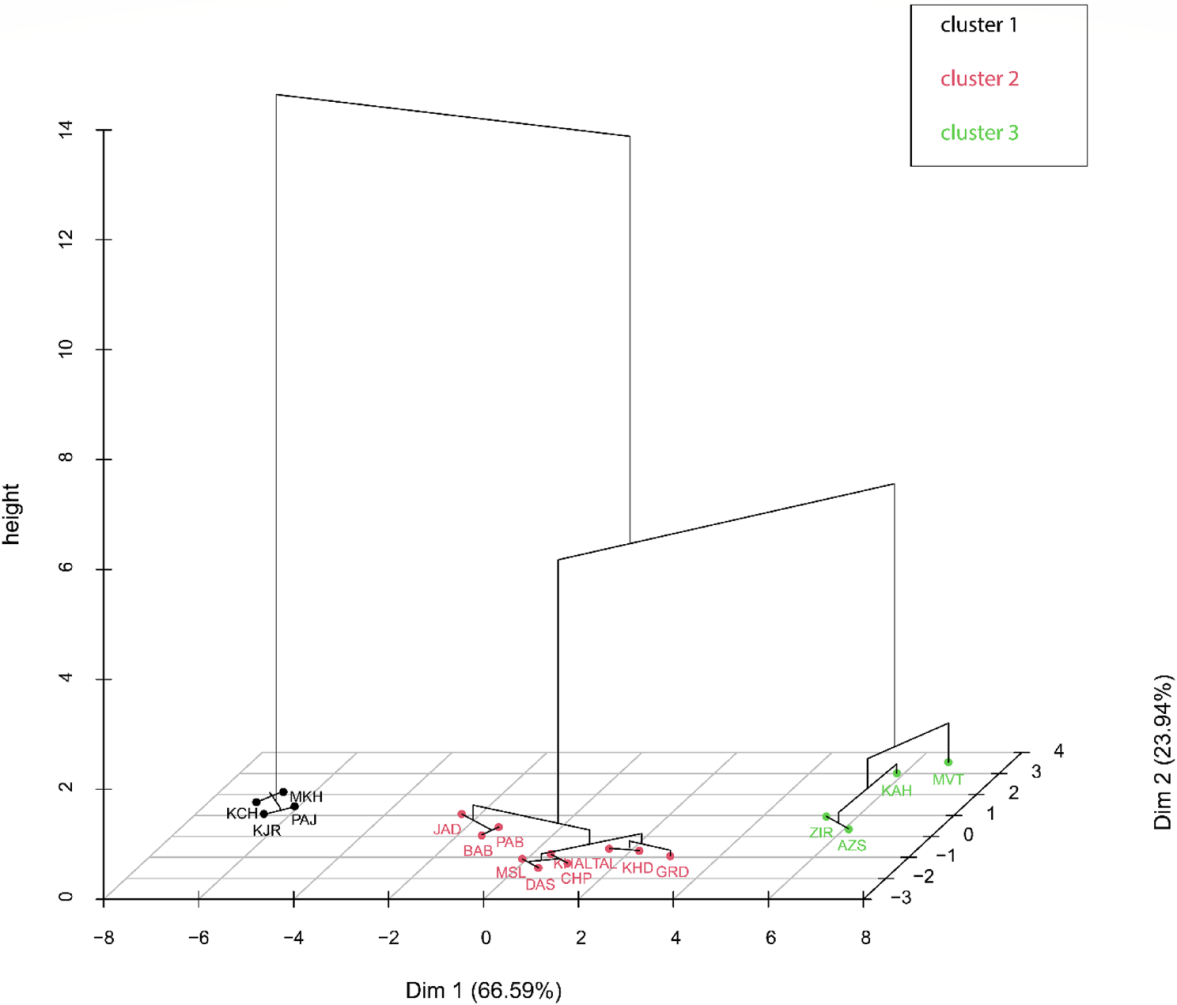
Hierarchical Clustering on Principal Components (HCPC) based on phenotypic and biochemical traits of 18 *O. mascula* natural populations. Dimension 1 explained 66.59% of the phenotypic variation, while the second dimension was only 23.94%. The populations clustered into three groups reflecting the influence of land cover types. For an explanation of abbreviations, see Tables 6 and 7.

The HCPC provides information on the factor map resulting from principal component analysis based on phenotypic and biochemical traits of the populations with a three-dimensional hierarchical tree, thereby providing a clearer view of the clustering pattern (Fig. 2). Dimension 1 explains 66.59% of the phenotypic variation, while the second dimension stands for only 23.94%. Results indicate three clusters that are projecting distinct positioning of the populations.

**Figure 3:**
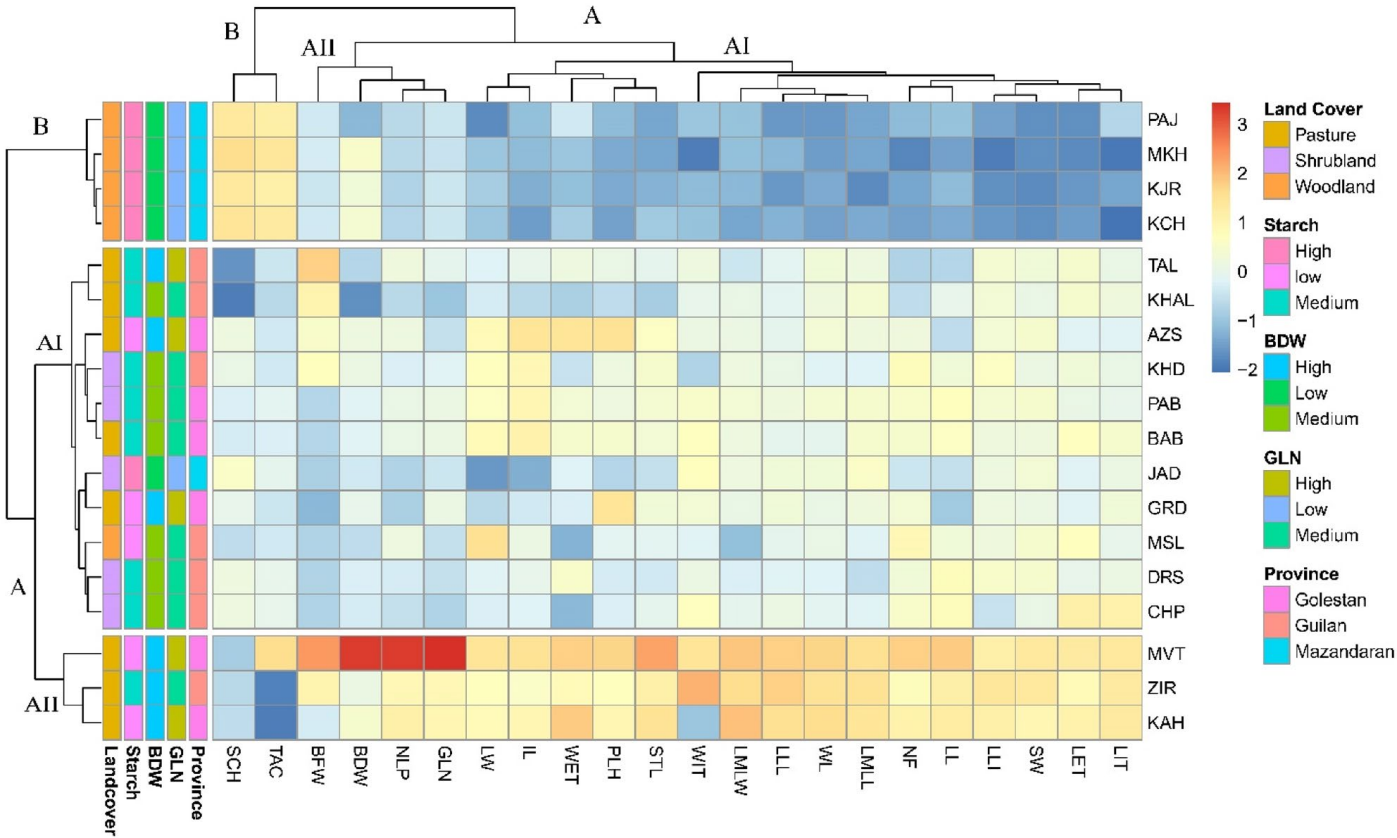
Heatmap visualization of two-way cluster analyses for 19 phenotypic and 3 biochemical traits (row tree) and 18 natural populations (column tree) evaluated in the growing season of 2020 in northern Iran, mainly Hyrcanian ecoregion. Column annotations indicate grouping based on 18 populations, 3 provinces, bulb dry weight, GLN, starch, and spike length. For an explanation of abbreviations, see Tables 6 and 7.

Two-way cluster analysis (heatmap) identified two main groups for phenotypic and biochemical traits (Fig. 3, column). Among biochemical traits, starch content and TAC formed a separated cluster (B) while the rest were placed in A. Traits such as GLN, number of leaves/plant (NLP), BFW, and BDW were grouped in a subcluster, and floral and leaf traits placed in AII. Two main clusters appeared at the population level (Fig. 3, row); cluster A included two subclusters; cluster AI encompassed populations from Mazandaran, Golestan, and Guilan provinces. However, populations in the subclusters were grouped based on similarity in their phenotypic or biochemical similarities, for instance, Talesh (TAS), Khalkhal (KHAL), Masouleh(MSL), Darestan (DRS), Chapoul (CHP), Khasib-Dasht (KHD) and Garmab-Dasht (GRD) all from Guilan province, clustered together. The three populations from the eastern side (MVT, KAH, and ZIR) stand out by forming a separate subcluster (AII). Four populations of Palamjan (PAJ), Mirkhamand (MKH), Kalech (KCH), and Kjour (KJR) with the lowest values of traits placed in the second cluster (B). Although discrepancies were observed in grouping patterns, clustering mainly succeeded in grouping the population according to their location or traits based on their identity (i.e., biochemical, tuber characteristics, leaves, floral features, etc.).

### Relationship between phenotypic and chemotypic traits and climatic variables

**Figure 4:**
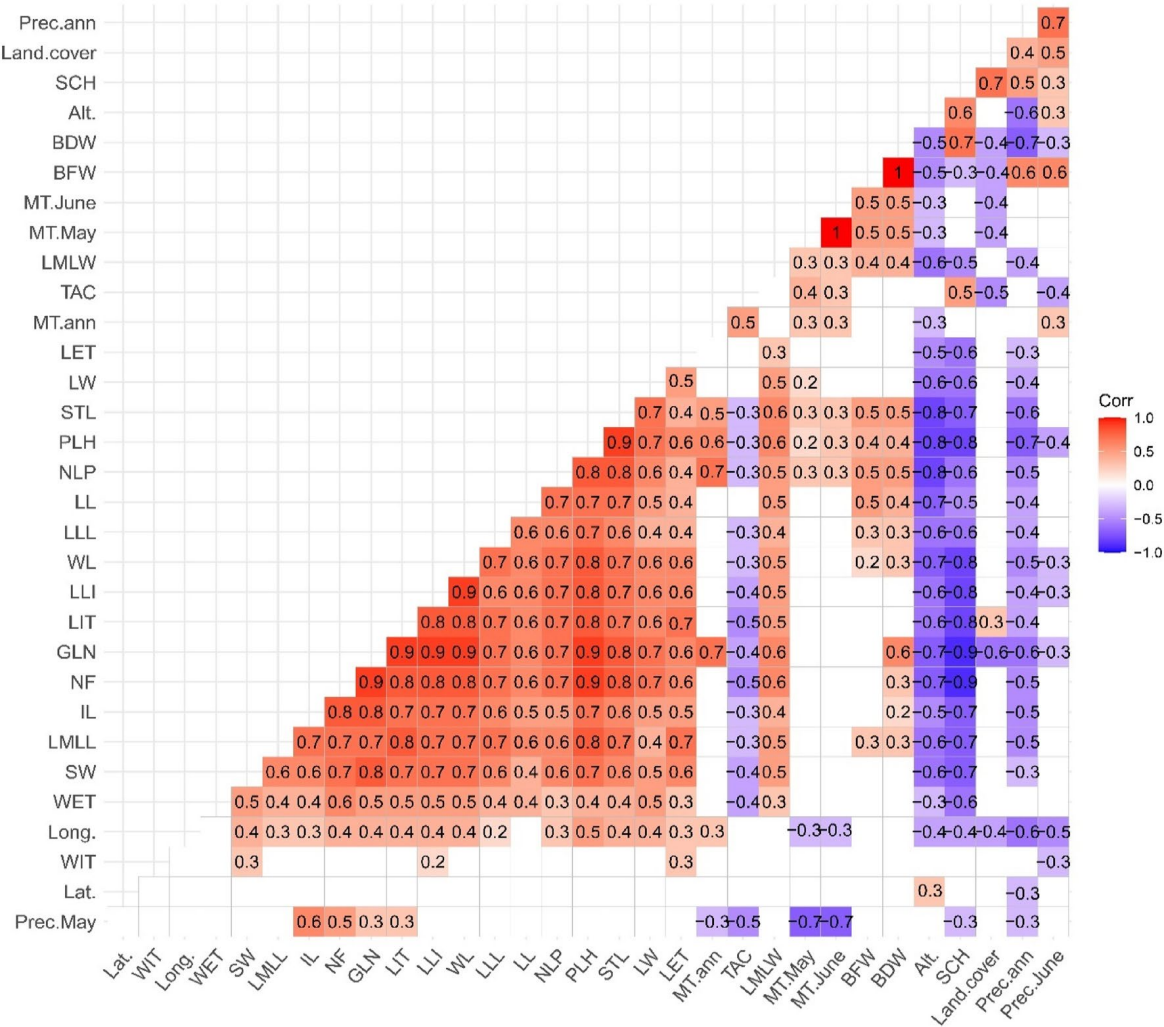
Correlogram of 19 phenotypic and 3 biochemical traits with 9 environmental and climatic variables evaluated in 18 *O. mascula* populations in northern Iran, Hyrcanian ecoregion. For an explanation of abbreviations, see Tables 6 and 7.

Correlation analyses revealed that the phenotypic traits negatively correlated with the annual precipitation and geographical region altitude and positively correlated with longitude. The important traits, PLH, number of flower (NF) and NLP responded positively to annual mean temperature (r = 0.7 and 0.6, *P* < 0.01), the same applies to GLN (r = 0.7, *P* < 0.01) and weakly with longitude. BWF and BDW weakly and negatively correlated to land cover (r =  − 0.4), but there was no correlation between land cover and other phenotypic traits (Fig. 4). The starch content significantly correlated with land cover and geographical altitude (r = 0.7, r = 0.6; *P* < 0.01) and weakly with annual precipitation (r = 0.5). Also, it was negatively correlated with longitude (r =  − 0.4) and showed no correlation with annual mean temperature. However, the GLN content significantly decreased with the increase in geographical altitude, annual precipitation, and land cover (r =  − 0.7, − 0.6, − 0.6, *P* < 0.01), respectively. A seesaw correlation was unveiled between GLN and starch (r =  − 0.9, *P* < 0.01), as observed earlier in Table 2 and Fig. 1. The former compounds correlated weakly with the increase of TAC, and the latter indicated a relatively positive relationship.

### Population genetics

#### Genetic diversity and differentiation of *O. mascula *populations

At the population level, TAS, MSL, and CHP had the highest observed alleles (*Na* = 2), while AZS had the lowest value (1.65) among populations with an average of 1.89. The highest effective alleles (*Ne* = 1.64) were related to the TAS population, and the lowest *Ne* (1.21) belonged to the ZIR population with an average of 1.46 (Table 3). Among the eighteen studied populations, the average polymorphism percentage (*P*%) was 31.14%, while MVT from Golestan had the highest (65.63%), and PAJ was the lowest (18.75%). With an average of 0.35, the highest value of Shannon's information index (*I*) was observed in the JAD population (0.49), while MSL and PBD populations (0.25) had the lowest level. An average of 0.42 expected heterozygosity (*He*) and 0.30 for Nei's gene diversity index (*H*) were observed. The parameters of *He* and *H* were the highest in the KAH population (0.55, 0.39, respectively), and the MSL population (0.31, 0.18, respectively) showed the lowest. The average of the populations' total genetic diversity (*Ht*) was 0.37, and the genetic diversity within populations (*Hs*) was 0.24.

**Table 3:** Genetic diversity statistics of 18 populations of *Orchis mascula* based on AFLP.

Population	*Na*	*Ne*	P%	*I*	*He*	*H*	*Ht*	*Hs*	*G’*_*st*_	*Nm*
TAS	2	1.64	27.84	0.37	0.39	0.25				
KHAL	1.94	1.48	38.07	0.31	0.41	0.23				
MSL	2	1.55	23.58	0.25	0.31	0.18				
DRS	1.93	1.63	25.57	0.41	0.42	0.26				
CHP	2	1.61	20.17	0.35	0.46	0.31				
KHD	1.99	1.51	25.85	0.29	0.41	0.27				
GRD	1.88	1.45	31.82	0.36	0.44	0.30				
JAD	1.89	1.55	22.44	0.49	0.48	0.3				
PAJ	1.99	1.49	18.75	0.39	0.39	0.25				
KJR	1.97	1.51	20.74	0.39	0.43	0.27				
KCH	1.95	1.43	22.44	0.42	0.46	0.29				
MKH	1.98	1.52	38.64	0.28	0.45	0.32				
PBD	1.88	1.32	28.98	0.25	0.46	0.31				
BAB	1.76	1.34	30.68	0.37	0.51	0.33				
KAH	1.73	1.36	**44.03**	0.29	**0.55**	**0.39**				
ZIR	1.67	1.21	31.25	0.35	0.52	**0.37**				
AZS	1.65	1.33	**44.03**	0.42	0.49	**0.35**				
MVT	1.69	1.38	**65.63**	**0.46**	0.52	**0.36**				
**Average**	**1.89**	**1.46**	**31.14**	**0.35**	**0.42**	**0.3**	**0.37**	**0.20**	**0.35**	**0.46**

Observed number of alleles (*Na*), the effective number of alleles (*Ne*), Shannon's diversity index (I), expected heterozygosity (*He*), Nei's gene diversity index (*H*), total gene diversity (*Ht*), gene diversity within populations (*Hs*), coefficient of genetic differentiation (*G’*st = (*Ht-Hs*)/*Ht*), gene flow (*Nm*). Significant values are in bold.

**Figure 5:**
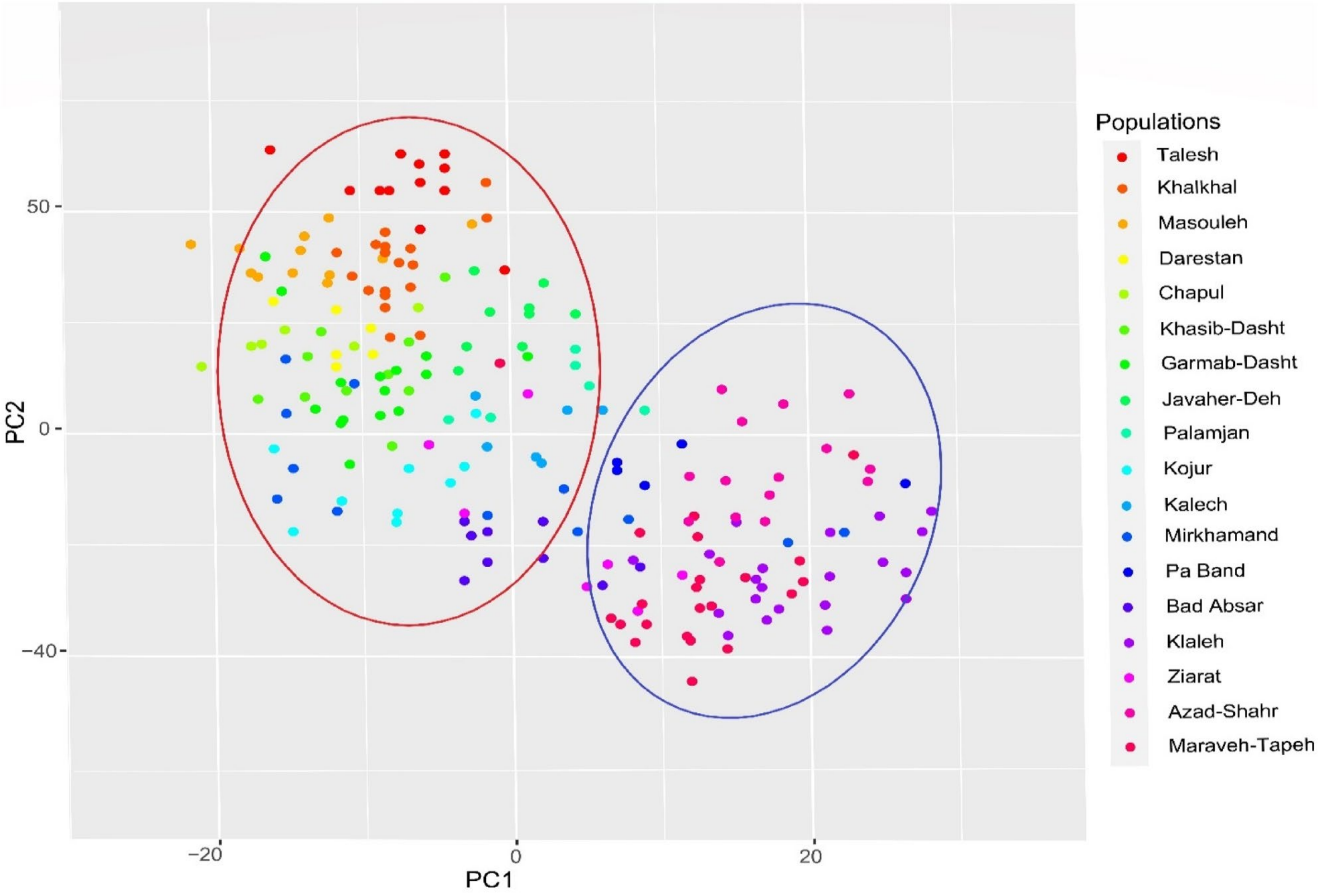
Principal coordinates analysis of 198 individuals of 18 populations of *Orchis mascula* based on the genetic similarity matrix derived from AFLP markers divided the populations into two groups. For an explanation of abbreviations, see Table 6.

The coefficient of genetic differentiation among populations (*G'*_st_) was 0.35, indicating that 35% of the genetic variation was among the populations, and 65% was within the populations. The *G'*_st_ was higher than average, considering that *G'*_st_ between 0.05 and 0.15 is defined as moderate and values over 0.30 as high^37^. Thus, *G'*_st_ reflected the significant genetic differentiation among the populations. These findings were consistent with the relatively low gene flow value (*Nm* = 0.46) observed among populations (Table 3). The principal coordinate analysis (PCoA) for 198 individuals of *O. mascula* revealed the presence of two groups (Fig. 5). Almost all 118 individuals of populations from Guilan (7 populations) and Mazandaran (5 populations) gathered into group I, and group II contained 80 individuals from 6 populations of Golestan. The pattern observed in PCoA confirms the clustering pattern revealed in heatmap visualization of two-way cluster analyses. Analysis of molecular variance (AMOVA) showed that value for genetic diversity occurred among populations (PhiPR = 0.388, *p* = 0.001) with a significant share of total genetic diversity (48%), whereas a larger proportion of variation (52%) existed within populations (PhiPT = 0.509, *p* = 0.001; Table 4). Differentiation among the three regions accounted for 20% of the genetic variance that existed among the three regions (PhiRT = 0.374, *p* = 0.001).

**Table 4:** Result of analysis of molecular variance (AMOVA) for 18 natural populations of *Orchis mascula*.

	Source	d.f	Sum of squares	Variance components	% of variation	Phi statistics	*P* value
Three types of Land cover	**Among groups**	2	1735.907	10.171	20	**PhiRT: 0.347**	0.001
	**Among Pops**	15	2964.190	16.076	31	**PhiPR: 0.388**	0.001
	**Within Pops**	180	4555.086	25.306	49	**PhiPT: 0.509**	0.001
	**Total**	197	9255.182	51.553			

Significant values are in bold.

#### Population structure

**Figure 6:**
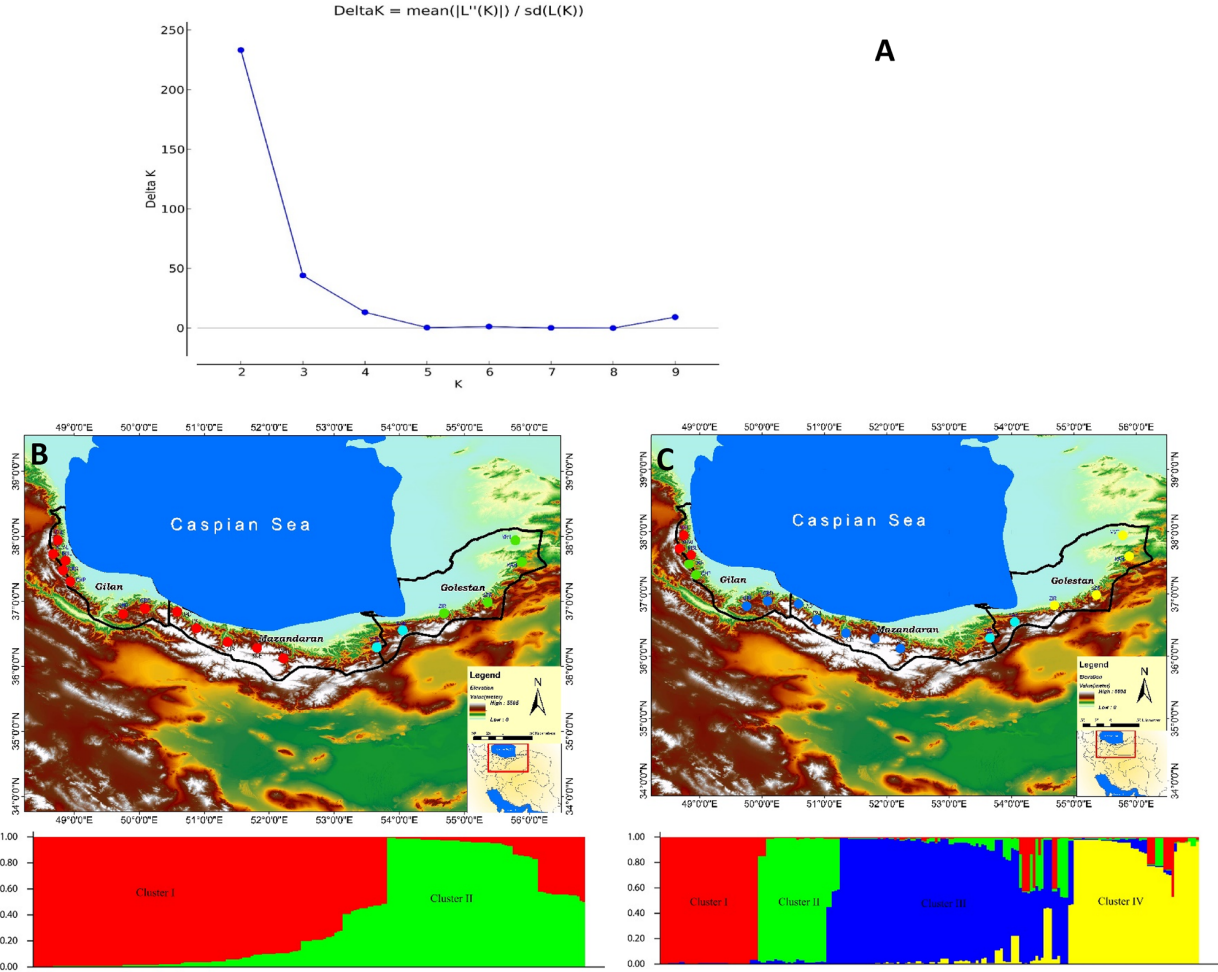
Map of 18 populations of and Bayesian admixture proportions identified by STRUCTURE of 198 individual plants of *Orchis mascula*. (**A**) The optimum number of subpopulations was determined using ΔK in the Bayesian clustering method, *K* = 2; (**B**) K = 2 and (**C**) *K* = 4. The colors used in the maps are approximately associated with STRUCTURE clusters. Cyan-colored circles represent the 'admixed' populations. For an explanation of abbreviations, see Table 6.

**Figure 7:**
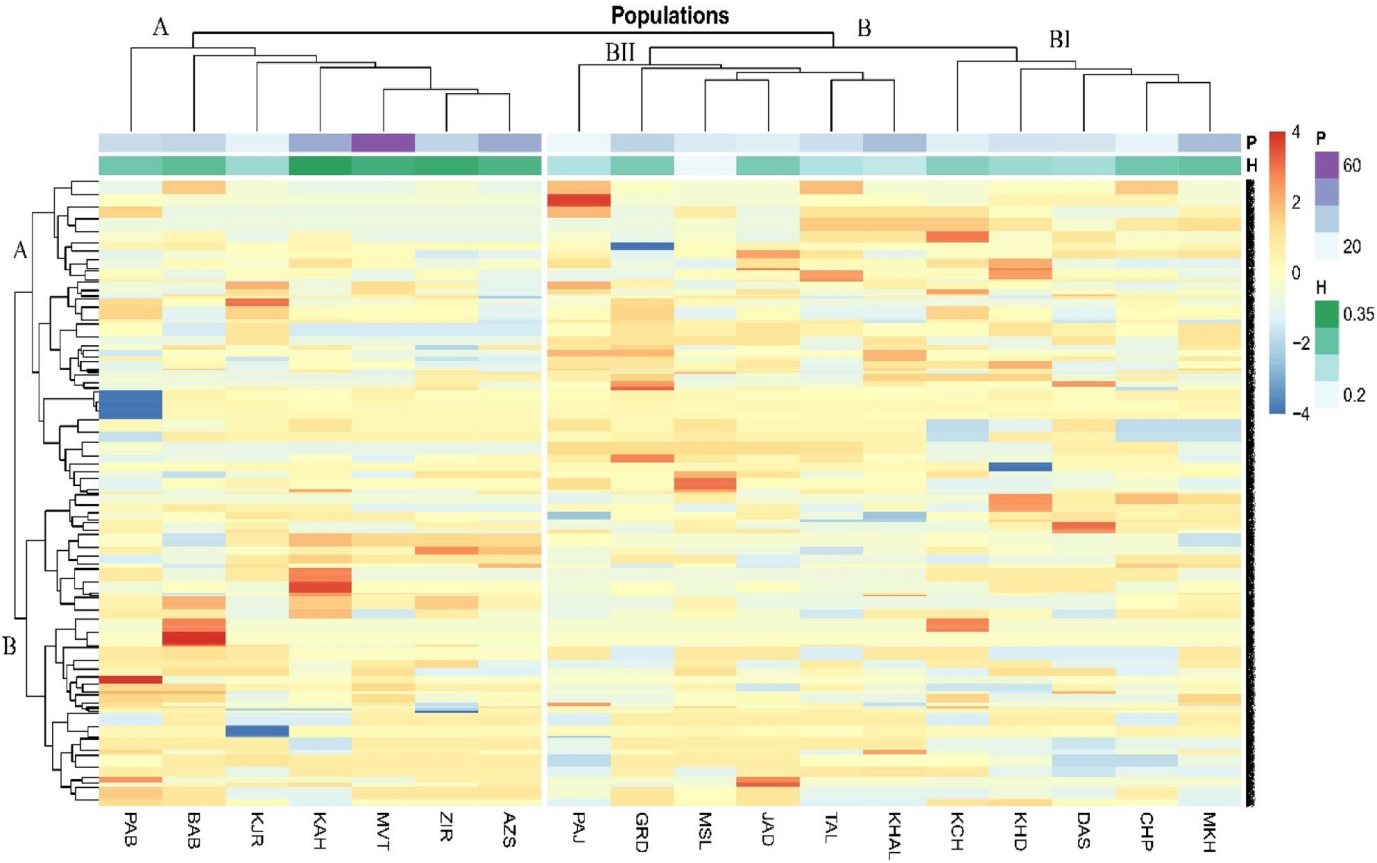
Heatmap visualization of two-way cluster analyses for 18 natural populations of *Orchis mascula* (row tree) and alleles generated by AFLP markers (column tree) evaluated in the growing season of 2020 in north of Iran, Hyrcanian ecoregion. For an explanation of abbreviations, see Tables 3 and 6.

Structure analysis divided the populations into two clusters at K = 2 (Figs. 6 and 7a), Cluster I, and Cluster II (Fig. 7b). Cluster I contained 117 individuals, among which 75 were from Guilan province and 42 individuals were from Mazandaran province (Fig. 7a, red). Cluster II included 63 individuals from Golestan province (Fig. 7a, green). However, 18 Golestan and Mazandaran individuals were identified as genetic admixtures (Fig. 7b, cyan). Structure analysis was further evaluated at K = 4 to see the consistency of the populations' membership, which changed to some level (Fig. 7c); Cluster I included 33 individuals, and 100% of their members came from the Guilan province. Cluster II had 24 individuals from Guilan province. Cluster III comprised 57 individuals, of which 15 were from Guilan and 42 from Mazandaran. However, this cluster had 24 individuals who shared some of them with Cluster IV (57 individuals), all belonging to Golestan populations. The admixture individuals were at the east of Mazandaran, more toward Golestan province (Fig. 7c, cyan color).

#### Influence of environmental variables on genetic diversity

**Figure 8:**
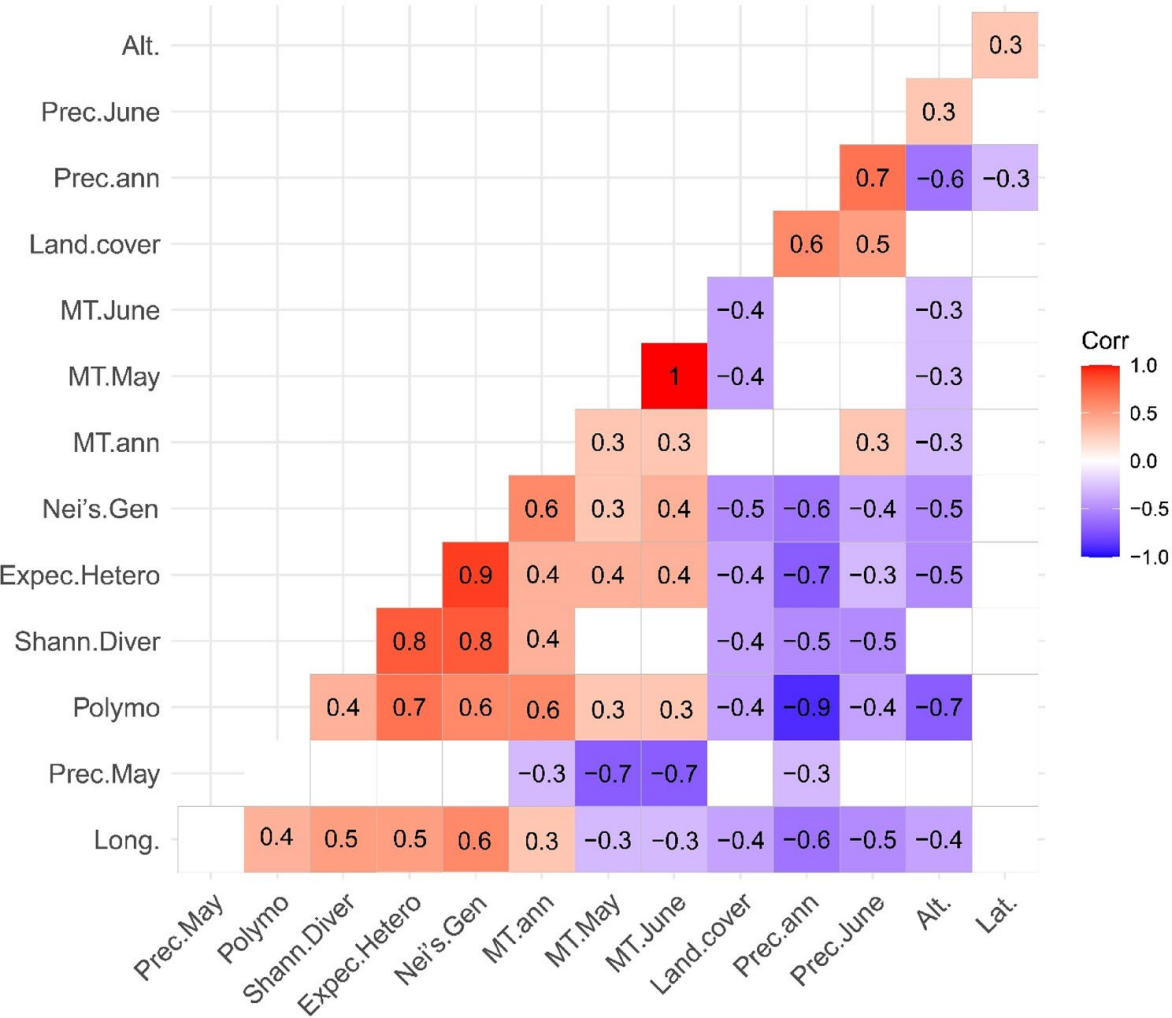
Correlogram of genetic diversity indices and geoecology variable. Abbreviations: precipitation in May and June (Prec. June, Prec.May); Polymorphism (%; Polymo.); Shannon Diversity Index (Shann.Diver); Expected heterozygosity (Expec.Hetero); Nei's genetic diversity (Nei's.Gen); Mean Temperature annual (MT.ann), in May and June (MT.June and MT.May); Annual precipitation(Prec.ann); Altitude(Alt.), Longitude(Long.). For an explanation of other abbreviations, see Table 3.

Correlation analyses revealed that population polymorphism significantly decreased with the increase of annual precipitation and geographical altitude (r =  − 0.9, − 0.7, *p* < 0.01, respectively) (Fig. 8), which suggested a pattern of low genetic diversity at high precipitation and elevation. The population polymorphism weakly and negatively correlated to land cover (r =  − 0.4), it also weakly and positively correlated to longitude (r = 0.4) and strongly to annual mean temperature (r = 0.6, *p* < 0.01). The population Nei’s genetic diversity demonstrated a negative correlation to annual precipitation (r =  − 0.6, *p* < 0.01), and positively to longitude (r = 0.6, *p* < 0.01). This parameter also potently and negatively correlated to land cover (r =  − 0.6, *p* < 0.01), precipitation in June (r =  − 0.4) and geographical altitude (r =  − 0.5). The population expected heterozygosity also significantly correlated to annual precipitation (r =  − 0.7, *p* < 0.01), negatively correlated to geographical altitude (r =  − 0.5), precipitation in June (r =  − 0.3), land cover (r =  − 0.4), positively with longitude (r = 0.5), annual mean temperature (r = 0.4), mean temperature in May (r = 0.4) and mean temperature in June (r = 0.4) (Fig. 8).

In testing for isolation by distance (IBD), the relationship between geographical and genetic distance and geographic and phenotypic distance were insignificant (Table 5). These results indicate the lack of effect of IBD. On the contrary, in isolation by environment (IBE), relationships between genetic and phenotypic distances with environmental variables were revealed to be significant, showing the influence of essential variables such as mean temperature or annual precipitation on controlling the variation among the populations. Other tests also exhibited significant correlations between phenotypic and genetic distance. For example, in other partial Mantel tests, the correlation between residuals of genetic × geographic with phenotypic distance or phenotypic × genetic with geographic was nonsignificant. The dominant effect of the environment was observed in all the comparisons (Table 5).

**Table 5:** Correlations between Nei's genetic distance, phenotypic distance, climatic (19 environmental variables), and geographic (km) differences among 18 populations of *Orchis mascula* tested with simple and partial Mantel tests for IBD and IBE.

Simple Mantel tests	*r*	*P*-value
Genetic × Geographic	0.112	n.s
Genetic × Environment	**0.556**	0.001
Phenotypic × Geographic	0.159	n.s
Phenotypic × Environment	**0.531**	0.001
Phenotypic × Genetic	**0.492**	0.013
**Partial Mantel tests**		
Genetic × Geographic | Phenotypic	0.098	n.s
Genetic × Environment | Phenotypic	**0.506**	0.001
Phenotypic × Genetic | Geographic	0.269	n.s
Phenotypic × Environment | Genetic	**0.518**	0.001

Significant values are in bold.

#### Marker-trait association

Applying two models of MLM1 and MLM2 to find a possible marker-trait association (Table S1) indicated strong comparisons of traits vs. AFLP markers with *r*^2^ > 0.40 at *P* < 0.01, where three of the markers revealed a simultaneous association with several important traits. For example, marker P-CCA + M-AGA-49 showed significant simultaneous associations with quantitative traits PLH, STL, NLP, LL, IL, NF, LLI, LMLL, BFW, and BDW on both MLM1 and MLM2 models (Table S1). Marker P-TGG + M-CTT-33 had concurrently significant associations with quantitative traits LW, IL, NF, LET, LIT, LLI, LMLL, LMLW, and biochemical traits GLN and TAC based on MLM1; and showed significant associations with quantitative traits of STL, LL, LW, NF, LET, LIT and biochemical trait GLN on MLM2 model. In addition, primer E-AGG + M-CGT-22 showed significant associations with quantitative traits PLH, IL, NF, WL, LMLL, and biochemical traits GLN and TAC based on MLM1. It showed significant associations with quantitative traits of PLH, SL, NLP, IL, NF, LLI, WL, and biochemical traits GLN and TAC based on MLM2 model (Table S1).

## Discussion

### Phenotypic plasticity in the context of environmental heterogeneity

It's often asserted that relying on phenotypic markers for genetic diversity assessment is an inferior approach given the significant influence of environmental variables^38–40^. While this is to some extent true, phenotypic traits are still valuable tools to unmask the responsiveness of the species across climatic gradients. Such information can reflect the fundamental pattern for species biodiversity conservation and find agronomically important traits with possible application in crop improvement programs. In this study, we examined the effects of environmental factors on genetic diversity and functional traits in *O. mascula*. To uncover the impact of sources of variation at the population level on phenotypic plasticity, an unbalanced REML mixed-model ANOVA was employed, showing the notable influence of two factors, land cover and the population (Table 1).

In this study, we observed a strong pattern of phenotypic response to environmental variables, revealing the importance of land cover type and precipitation. *O. mascula* is relatively tolerant to shade; however, deep shade can significantly jeopardize its survival. Several comprehensive studies have used this species to understand the influence of light availability on reproduction performance^41,42^. The dominant pattern witnessed in this study was the better performance of *O. mascula* in pasturelands/grasslands of east Hyrcanian, which could be linked to the availability of light and soil nutrients (i.e., not content) as opposed to the woodlands of Mazandaran and low altitudes of Guilan. The determinative effect of light was studied by^41^, who found that the overall growth rate was higher in coppiced than undisturbed woodland. Although plant species respond differently to shade, many usually do not succeed in flowering under deep shade^42^. In addition to light, higher nutrient availability in clear-cuts and pastures^43^ may have facilitated better growth performance.

High variation in floral traits, particularly in labellum length and width, has been reported recently in *Orchis purpurea* Huds.^44^, and in *O. mascula* and *O. pauciflora* Ten.^45^. The results of the current study were, to a large extent, consistent with these studies. In the comprehensive phenotypic study of Ebrahimi, et al.^26^. quantitative floral traits showed considerably higher variation than the results reported here; however, the number of flowers had the highest coefficient of variation (41.34%), similar to our results (62.07%, Table 1). Their study area was Abr Forest, where *O. mascula* manifested considerably higher variation among almost all qualitative and quantitative floral traits indicating high ornamental values in these populations.

Growth and the probability of flowering are in most tuberous orchids primarily associated with access to essential nutrients and the carbohydrates stored in the belowground organs^46,47^. Interestingly, two primary compounds in underground organs of *O. mascula* (GLN and starch) showed an inverse relationship (Fig. 1), similar to what has been previously reported by Tekinşen and Güner^48^ on several salep species, including *Orchis italica* Poir. and *Orchis morio* L.. The starch and GLN content reported on round-type tubers like *O. mascula* in Iran (6.15% and 22.1%, respectively)^22^ were notably lower than our observation (Table 2; 11.13 g/100 starch and 38.15 g/100 GLN). The dry matter produced in bulbs was considerably higher in populations in the east or at high elevations in the wast. High nutrient and light availability together with a higher amount of assimilated carbohydrates, can significantly contribute to better growth and reproduction. Nonetheless, in terrestrial orchids relying on stored material belowground may not be sufficient at the beginning of growth or reproduction when demand reaches its peak^49^. Improving local light conditions in woodland orchids by coppicing may directly or indirectly positively affect orchids^50^ and lead to larger plants with a higher flower number and larger inflorescence size. Similarly, the dry matter under the ground can result from the increased light conditions and explain why the difference in land cover significantly influenced plant performance in this study (Fig. 4).

Another major player shaping the observed patterns of phenotypic variation is precipitation, which clinaly decreased from 800 mm in the west of Hyrcanian to 250 mm in the east. The low rainfall may impose abiotic stress on *O. mascula* populations in Golestan and lead to increased phenotypic variation. It's possible that increased dry matter of tubers in areas with lower precipitation encourages the accumulation of carbohydrate compounds in *O. mascula*. Aggregation of carbohydrate compounds is a common drought tolerance strategy^51^. Low predictability of precipitation may contribute to a relative increase in floral trait variation on the eastern side. March-Salas, et al.^52^ reported enhanced within-individual and within-population variability in flowering phenology by less foreseeable precipitation patterns in *Onobrychis viciifolia* Scop. populations in a multigeneration experiment.

### Population genetics and structure

Preserving genetic diversity is crucial since it can buffer against upcoming short-term and long-term climate-change-driven stress, disturbance, inbreeding depression, and interactions with hostile species^2,53,54^. In situations where high genetic diversity can be a significant advantage, the population faces unpredictable levels of abiotic stress in the environment, such as high temperature or water stress^8^. Population genetic diversity is the reflection of mating system, habitat fragmentation, and climatic elements, which survival of plant species depends on as the greater genetic diversity is, the higher chance of coping with various climatic changes.

In this study, we found that genetic variation among *O. mascula* populations follows the pattern of environmental heterogeneity and is significantly affected by climatic variables such as temperature, precipitation, and land cover. Genetic diversity (*H*) at the population level with an average of 0.30 was observed to be higher than the previous studies on *O. mascula*; for example, Jacquemyn, et al.^41^ reported an average of 0.16 or Gholami, et al.^31^ 0.10. However, the latter used ISSR markers with a small sample size (5). Strong geographical affinity seemed to be followed by the pattern of genetic diversity among populations, analogous to phenotypic variation, the level of genetic diversity was notably higher in the populations of Golestan, where KLH had the highest *H* (0.39) and other populations from this province similarly showed high *H* (Table 3). As an orchid species with a mixed breeding system, *O. mascula* in this study was possibly affected by several factors. Land cover type appears to have been a significant player because the genetic variation among populations was considerably higher in pastureland/grassland populations. This response could be supported by the light availability of the eastern populations against woodland of the west and Mazandaran province; brighter conditions mean the prevalence of pollinators in addition to a higher number of flowers (as was observed in phenotypic diversity). Thus, the possibility of pollinators visiting more flowers available before leaving due to the food-deceiving nature of this species is higher; however, the gene flow (*Nm*) mean was low.

The genetic structuring of populations was strong in K = 2 and K = 4 (Figs. 6 and 7a), further supporting the high genetic differentiation (*G'*_st_: 0.35, Table 3). It could be argued that the large geographic distance from the west to the east of the Hyrcanian ecoregion (≈ 500 km) strongly contributes to the limited gene flow. However, this does not seem to be the case, as we found no evidence for IBD. On the other hand, the genetic structure mainly supports the prominent effect of the environment on shaping this pattern among populations. Low gene flow is often caused by habitat loss and fragmentation, leading to reductions in population size and increased spatial isolation^55,56^. Low within-population genetic diversity (52%) and high differentiation (*G'*_st_: 0.35) in this study are contrary to previous results reported on *O. mascula*, showing a high genetic diversity within population (92%) and low *G*'_st_ (0.125)^41^. Moreover, the woodland type did not affect genetic partitioning. This is in sharp contrast with the results of this study, where 20% percent of the total genetic diversity was partitioned among three groups of land covers (woodland, shrubland, and pastureland). Differences in scale can explain these contrasting results. Whereas Jacquemyn, et al.^57^ studied 15 populations in the local landscape with slight variation in climatic conditions, in our study, populations were studied across a much larger scale leading to strong differences in climatic conditions between eastern and western populations and significant IBE. This suggests that populations have genetically adapted to the different precipitation regimes across Hyrcania and that this genetic adaptation has contributed to the observed high genetic differentiation. These results further support the findings of Siepielski, et al.^58^, who showed that precipitation was a dominant driver of natural selection in plant and animal populations for over 150 species.

### Implication for breeding efforts

Given its economic importance, *O. mascula* population sizes have been reduced beyond recovery in many areas in the west, northwest, and north of Iran. Developing a breeding program could be a sustainable option to counteract the further loss of populations. Selection of the most suitable populations and cultivation of local plant material could represent the first steps in a sustainable management of *O. mascula* populations in the wild. Our results showed that populations from Golestan possessed several important desirable traits, including a high GLN content and bulb dry weight that meets the high-quality threshold^48^. Another approach to probe for favorable traits is the percent of variation (%var) populations display^59^. These traits mentioned above also had the highest variation percentage under the effects of land cover and population (Table 1). A phenotypic study on *O. mascula* populations by^26^ also indicated high variation for tuber characteristics, BFW & BDW in particular.

Additionally, marker-assisted phenotypic trait selection may provide heritable traits with high economic value. In this context, marker-trait associations based on MLM1 and MLM2 models revealed significant associations of P-CCA + M-AGA-49 with important traits such as PLH, BFW, and BDW, P-TGG + M-CTT-33 and E-AGG + M-CGT-22 with PLH and GLN (Table S1). The associated traits observed here are important in the adaptability of *O. mascula* as they were found to be most responsive in the populations coping with stressful conditions. These linked markers with another quantitative trait might also show a pleiotropic effect or may be located very closely^60^. Marker-trait association in terrestrial orchids, including *O. mascula*, has been previously reported by Gholami, et al.^31^, who also observed a significant association between ISSR markers and tuber dry weight. Notable traits with high potential for selection have been identified in this study. However, it should be noted that while all measurements were conducted on samples collected from natural populations, a common garden experiment can provide better insights into this trait relationship and its reliability for further application.

Our study illustrates the functional trait variation in response to environmental variables in relation to genetic diversity, offers empirical evidence that supports a combination of different datasets in population-level studies, and observes the interactions from a broader perspective to gain a more reliable estimation. Readily measurable functional traits and their incorporation with population genetics provide a deeper understanding of species' ability to adapt to environmental changes and facilitate using this variation for applications such as plant breeding^61^. Our findings in *O. mascula* can be used as the foundation of a plant breeding program that allows for the cultivation of *O. mascula* in an agricultural setting, thereby reducing the demand for wild-collected tubers. Given the current limitations of the legal protection provided for *O. mascula,* this may be the best way to conserve natural populations of this species, as well as other species of tuberous orchids.

## Material and methods

### Study sites and sampling

In the spring of 2020, eighteen populations of *O. mascula* were sampled in the Hyrcanian ecoregion (Table 6), following Flora Iranica and Flora of Iran^62,63^. This is an exceptionally rich biodiversity area containing relict species in Tertiary period forests covering extensive regions between Iran and Azerbaijan. Anthropogenic threats to wild species in these areas include exploitation of natural populations and habitat destruction through the construction of housing and industrial activity. It should be noted that in 2020 accessing the populations of *O. mascula* for this study was possible because graduate researchers are automatically allowed to pursue research within their field of study, exempt from common regulations to collect plant material unless the plant of interest is in the protected, disputed areas near borders or the collection is for commercial purpose. In this case, our study was a conservation/breeding attempt entirely in line with the current rules to access endangered plant species; thus, official permission or license was not needed. However, in Jul. 2021, new regulations were announced (the complete list can be found at https://rc.majlis.ir/fa/law/show/1667090) in which access to any wild population requires official permission. Dr. Gholizadeh identified the species identity of the samples, and voucher specimens from the populations were deposited in the University of Guilan Herbarium (GUM; no. 5631 to 5649), available for botanical studies with an official request.

**Table 6:** Details of geographical locations where populations of *Orchis mascula* were sampled.

Populations	Sample no	Latitude (°N)	Longitude (°E)	Altitude (m)
**Guilan**				
Talesh(TAS)	12	37° 53′ 48.95″ N	48°44′55.37″ E	1432
Khalkhal(KHAL)	18	37° 59′ 9.93″ N	48° 28′ 2.86″ E	1888
Masouleh(MSL)	12	37° 19′ 22.07″ N	48° 57′ 23.32″ E	1275
Darestan(DAR)	6	36° 58′ 11.24″ N	48° 55′ 37.56″ E	1113
Chapul(CHP)	6	37° 8′ 30.17″ N	49° 9′ 32.94″ E	752
Khasib-Dasht(KHD)	9	36° 56′ 46.81″ N	50° 4′ 1.99″ E	1976
Garmab-Dasht(GRD)	12	36° 53′ 4.28″ N	50° 13′ 1.44″ E	697
**Mazandaran**				
Javaher-deh(JAD)	9	36° 51′ 46.56″ N	50° 27′ 20.62″ E	2632
Palamjan(PAJ)	6	36° 45′ 44.12″ N	50° 30′ 58.92″ E	1852
Kojur(KJR)	9	36° 22′ 50.82″ N	51° 45′ 57.01″ E	1609
Kalech (KCH)	6	36° 16′ 50.21″ N	51° 39′ 38.44″ E	2841
Mirkhamand(MKH)	12	36° 19′ 7.95″ N	51° 53′ 19.53″ E	2513
**Golestan**				
Pa Band(PBD)	6	36° 32′ 43.74″ N	54° 11′ 51.16″ E	2208
Bad Absar(BAB)	9	36° 26′ 3.46″ N	53° 55′ 36.74″ E	1284
Klaleh(KAH)	21	37° 42′ 0.83″ N	55° 57′ 31.31″ E	830
Ziarat(ZIR)	9	36° 40′ 27.36″ N	54° 30′ 4.76″ E	1538
Azad-Shahr(AZS)	15	37° 6′ 16.54″ N	55° 23′ 7.23″ E	1845
Maraveh-Tapeh(MVT)	21	37° 51′ 25.82″ N	55° 33′ 19.18″ E	394

A total of 198 individuals from 18 populations, with at least 20 m distance between individuals in each population, were collected from these populations. Nineteen quantitative phenotypic traits related to the size of the plants and flowers (Table 7) were recorded for each individual. A digital caliper was used to measure the quantitative traits with a precision of 0.01 mm. Using a digital scale, the fresh weight of bulbs was recorded, and subsequently, bulbs were dried in the oven at 72 °C and measured for dry weight afterward.

**Table 7:** Characteristics used in the phenotypic and biochemical analyses of *Orchis mascula* populations.

Quantitative traits	Biochemical traits	Primer sequences 5' → 3'
1. Plant height (PLH; cm)	Glucomannan content (GLN; g/100 g)	P-TGG + M-CTT
2. Stem length (SL; cm)	Starch content (g/100 g)	P-TGG + M-TTG
3. Number of leaves/plant (NLP)	Total content of anthocyanin (mg/gr) (TAC)	P-CCA + M-AGA
4. Leaf length (LL; cm)		P-GCA + M-CTC
5. Leaf width (LW; cm)		P-GCA + M-AGT
6. Inflorescence length (IL; cm)		P-ACC + M-AGA
7. Number of flowers (NF; cm)		P-GTT + M-CTT
8. Length of external tepals (LET; cm)		P-CCA + M-CTT
9. Width of external tepals (WET; cm)		P-GTT + M-ATC
10. Length of internal tepals (LIT; cm)		P-ACC + M-AGT
11. Width of internal tepals (WIT; cm)		E-AAC + M-CTT
12. Length of lip (LLI; cm)		E-AAC + M-TGT
13. Width of lip (WL; cm)		E-AAC + M-CCA
14. Length of median the lobe of the lip (LMLL; cm)		E-AAG + M-TGT
15. Length of Median lobe width of the lip (LMLW; cm)		E-ACA + M-AAG
16. Length of the lateral lobe (LLL; cm)		E-ACC + M-CAG
17. Sidelobe width (SW; cm)		E-AGA + M-CAG
18. Bulb Fresh weight (BFW; g)		E-AGC + M-CGA
19. Bulb Dry weight (BDW; g)		E-AGC + M-CTT
		E-AGG + M-CGT

List of primers' combinations used in this study is also presented in the last column.

Young and fresh leaves were collected from each individual, immediately dried with silica gel, and transported to the laboratory for DNA extraction. For each population, the land cover was characterized by assessing the percentage of vegetation (e.g., woodland, shrubland, and pastureland/grassland). Using Google Earth Pro®, high-resolution aerial images of the locations were acquired. The interpretation of the images was based on visual classification using the ArcGIS 10.4® software (URL: https://www.esri.com/en-us/arcgis/products/arcgis-desktop/resources), a program used to calculate the areas and percentages of land use and land cover by each category.

### Chemotyping

#### Glucomannan and starch contents

To produce salep powder from bulbs, a traditional method previously described by Tekinşen and Güner^48^ was used. For this purpose, the bulbs were washed with cold water, cleaned from dirt and mud, and boiled for 10–15 min in milk; then, the samples were dried at 21 ± 2 °C until they hardened (7–10 days). The dried specimens were first cut into small pieces and then powdered into flour by milling. Further analysis was carried out to measure the components of interest in the flour.

The samples were prepared according to procedures specified for GLN and starch using GLN (K-GLUM 10/04) and total starch (AA/AMG 11/01, AOAC Method 996.11) assay kits (Megazyme International Ireland Limited, 2004a, b). The GLN and starch contents of the samples were determined by measurement of absorbance values of prepared blind and sample solutions (for GLN A_1_, A_2_, A_3_; for starch ΔA, F) in a UV–Vis Spectrophotometer (Shimadzu – UV Mini 1240) at 340 nm (for GLN) and 510 nm (for starch) and using the following formula 1 and 2, respectively^64,65^:

$$\begin{aligned} & \Delta {\text{A}}_{{{\text{glucomannan}}}} = \left( {{\text{A}}3{-}{\text{A}}1} \right)_{{{\text{sample}}}} {-}\left( {{\text{A}}3{-}{\text{A}}1} \right)_{{{\text{blank}}}} \times 36.8 \, \left[ {{\text{g}}/100\;{\text{g}}} \right] \\ & {\text{Starch}} = \Delta A \times \left( {{\text{F}}/{\text{W}}} \right) \times 90 \, \left[ {{\text{g}}/100\;{\text{g}}} \right] \\ \end{aligned}$$

where ΔA = absorbance value of sample solution compared with blind solution; F = 100 (μg of glucose control)/absorbance value of glucose control (1.03); and W = the weight of the sample (100 mg). The final content was reported as g/100 g of dry matter.

#### Total anthocyanin content

To measure tepals and labellum's anthocyanin content (purple pigment), the pigment was first extracted with 0.5 mL methanol/0.1% HCl for 24 h in the dark. Then the absorbance was read at 510 nm using a UV–Vis spectrophotometer (UV1601; Shimadzu, Kyoto, Japan)^66^. The anthocyanin content is expressed as mg per gram of fresh weight.

#### Genotyping

Genomic DNA was extracted from silica gel dried young leaves of the individuals utilizing CTAB procedure^67^. The quality and quantity of extracted DNA were assayed by 1% agarose gel electrophoresis and NanoDrop® spectrophotometer and diluted with sterile distilled water to give a final 100 ng/μL. The method described by Vos, et al.^68^ was employed with slight modification for AFLP analysis. An amount of 250 ng genomic DNA (per sample) was homogenized with EcoRI and MseI primers without a selective base in pre-amplification and with three selective bases in amplification in 20 µL master mix (2 µL Buffer, 2 µL 10 X BSA, 0.5 µL MseI, 0.5 µL EcoRI, 4.5 µL double-distilled water). The obtained combination of genomic DNA and restriction enzymes were incubated for 12 h at 30 °C. Then double-stranded EcoR I (Eco) and Mse I (Mse) linkers were ligated to the restriction fragments without additional nucleotides and afterward amplified by primers with three additional nucleotides at the 3′-end (Table 1). In selective amplification, the combinations of primers were used. The PCR Thermo cycles were carried out with a program of 35 three-step cycles, including 94 °C for 1 min, 56 °C for 30 s, and 72 °C for 1 min. On 6% acrylamide gel in vertical electrophoresis, Bio-Rad, the DNA fragments were separated, and to reveal the bands, the silver staining method was applied^69^. The banding pattern of the amplified DNA fragments was turned into binary codes as presence (1) or absence (0), and only the consistently repeatable bands were scored. Weak or smeared bands were excluded, and the fragments of the same molecular weight were counted as the same locus.

### Data analysis

#### Phenotypic data

A principal component analysis (PCA) was performed on the phenotypic data. The first two principal components were retained as they explained the highest portion of the total variation and were treated like traits and subjected to further analysis. To determine how phenotypic variation is partitioned between different types of land covers (fixed effect), among populations (random effect), and among individuals (error), an unbalanced restricted maximum likelihood (REML) mixed-model analysis of variance (ANOVA) was conducted for the 22 measured traits and PC1 and PC2 (Table 1). Means ± standard errors of the traits based on each population were also provided (Table 2). The analyses were implemented in PROC MIXED (SAS Institute, 2008, Cary, NC).

Hierarchical Clustering on Principal Components (HCPC) using "Factoextra" and "FactoMineR" packages^70,71^ was used to assess relationships among phenotypic traits. The phenotypic diversity expressed by the sampled *O. mascula* population was further explored with a two-way clustering analysis using the "Heatmaply" package in the R software^72^. Given the difference in the scaling of the traits, each trait was normalized using Z-score before cluster analysis.

A correlation analysis between phenotypic traits was carried out using Pearson's correlation coefficients (P 0.01) using the "CorrPlot" package^73^. The same package was utilized to assess the relationship between genetic diversity indices and environmental factors (average temperature, annual precipitation, and during the growing seasons, altitude, longitude, and latitude).

### Molecular data

#### Genetic diversity

Genetic variation indices including the observed number of alleles (*Na*), the effective number of alleles (*Ne*), expected heterozygosity (*He*), Shannon's diversity index (*I*), Nei's genetic diversity (*H*)*,* total heterozygosity (*Ht*), and intra-population genetic diversity (*Hs*) were computed using POPGENE v3.0^74^ (Table 3). All these calculations assumed that populations were in Hardy–Weinberg equilibrium.

#### Genetic structure

A Cluster (heatmap) analysis was performed at the population level based on the matrix of Nei's unbiased genetic distance using "ggplot2"^75^ and "Pheatmap"^76^ packages in the R software. A principal coordinate analysis (PCoA) was carried out using the "logisticPCA" package^77^ in the R software to visualize the spatial structure. Bayesian model-based cluster analysis using STRUCTURE v2.3.4^78^ was also used to infer genetic structure and define the number of clusters in the data set. The correlated allele frequencies and admixed model were applied with 50,000 burn-in and 100,000 MCMC. The assumed number of groups (*K*) varied from 1 to 10, and 10 runs per *K* were performed. The STRUCTURE HARVESTER^79^ determined optimum *K* based on L(*K*) and Δ*K*. The structure result at K = 2 and K = 4 were mapped according to geographical locations using the ArcGIS 10.4® software (URL: https://www.esri.com/en-us/arcgis/products/arcgis-desktop/resources).

#### Genetic differentiation

To assess the magnitude of genetic differentiation, the partitioning of total genetic variation at different scales was computed with analysis of molecular variance (AMOVA) in "vegan"^80^ in the R software. Shannon differentiation coefficient (*G'*_st_) was calculated based on the following formula, G*'*_st_ = (Hsp − Hpop)/Hsp (Hsp, total Shannon information index; Hpop, average Shannon information index within the population), and the gene flow (*Nm*) among populations was estimated using POPGENE v3.0^74^.

#### Isolation by distance/isolation by environment

To test the effects of geographic distance and environmental differences on the genetic and phenotypic structure, the genetic distances among populations were calculated using Nei's genetic distance implemented in R package "Poppr"^81^ and phenotypic distance using the "dist" function in R software. Euclidean geographic distances were calculated using the "fossil" package^82^. For sampling sites, climatic variables were collected from WorldClim version 2.067. Bioclimatic variables (19) were considered as different environmental space vectors and used the Canberra distance to calculate the distance between populations in this vector space. For isolation by distance (IBD) and isolation by environment (IBE), the relationship of genetic and phenotypic distances with geographic and environmental conditions was analyzed, respectively, using simple and partial Mantel tests implemented in the R package "vegan"^80^. Additionally, all other possible comparisons (geographic, phenotype, genetic, and environment) were tested.

#### Trait-marker association analysis

Two models were compared to test the marker-trait association between AFLP and phenotypic and chemotypic traits using TASSEL 4.0.1. (http://www.maizegenetics.net/bioinformat-ics) ^83^: a Mixed Linear Model (MLM) using the kinship matrix (K) estimated by the STRUCTURE HARVESTER^79^ as a random effect (MLM1) and an MLM using both Q and K (MLM2). Results were compared to determine a better model. Significance of associations between loci and traits was described as P-values (a probability level of 0.001).

## Supplementary Information

Supplementary Information.

## Data Availability

The datasets used and/or analyzed during the current study available from the corresponding author on reasonable request.
