# Supplementary material for: The impact of environmental and climatic variables on genetic diversity and plant functional traits of the endangered tuberous orchid (Orchis mascula L.)
Source: Sci Rep. 2022 Nov 17;12:19765. doi: 10.1038/s41598-022-19864-4 (PMC9672365; doi:10.1038/s41598-022-19864-4)
Supplement: Supplementary file 1 — Supplementary Information. [file 41598_2022_19864_MOESM1_ESM.docx]

**The impact of environmental and climatic variables on genetic diversity and plant functional traits of the endangered tuberous orchid (*Orchis mascula* L.)**

Mohammad Mafakheri^a^*, Mehdi Bakhshipour^b^, Mina Omrani^c,1^, Hamid Gholizadeh^d,1^, Najmeh Rahimi^e,1^, Ali Mobaraki^c^ and Mehdi Rahimi^f^

^a^Department of Plant Sciences, University of California - Davis, Davis, CA  95616, USA.

^b^Department of Horticultural Sciences, Faculty of Agricultural Sciences, University of Guilan, P.O. Box 41635-1314, Rasht, Iran

^c^School of Science and Technology, Faculty of Science, Agriculture, Business and Law, University of New England, Armidale, NSW 2351, Australia

^d^Department of Biology, Faculty of Basic Sciences, University of Mazandaran, Babolsar, Mazandaran, Iran

^e^Department of Chemistry and Biochemistry, New Mexico State University, Las Cruces, NM, USA

^f^Department of Biotechnology, Institute of Science and High Technology and Environmental Sciences, Graduate University of Advanced Technology, Kerman, Iran

*Corresponding author: mmafakheri@ucdavis.edu

^1^these authors contributed equally to this research.

**Supporting Information**

**Table S1.** Association of AFLP markers with phenotypic and biochemical traits of *Orchis mascula* population-based on the mixed linear model (MLM1 and MLM2).

| Traits | MLM1 | | | Traits | MLM2 | | |
| --- | --- | --- | --- | --- | --- | --- | --- |
|  | Marker | P value | R^2^ (%) |  | Marker | P value | R^2^ (%) |
| PLH | P-TGG+M-CTT-36 | 0.02903 | 0.45408 | PLH | **E-AGG+M-CGT-22** | **0.0097** | **0.57508** |
|  | P-TGG+M-CTT-37 | 0.03058 | 0.48364 |  | E-AAC+M-CTT-21 | 0.0108 | 0.50405 |
|  | **P-CCA+M-AGA-49** | **0.01105** | **0.82997** |  | P-TGG+M-CTT-36 | 0.03059 | 0.49439 |
|  | **E-AGG+M-CGT-22** | **0.00013** | **0.68326** |  | P-TGG+M-CTT-37 | 0.04606 | 0.49072 |
|  | P-GTT+M-CTT-28 | 0.0443 | 0.50079 |  | P-ACC+M-AGA-52 | 0.04991 | 0.49001 |
|  | P-GTT+M-CTT-22 | 0.01073 | 0.48412 | STL | **P-CCA+M-AGA-49** | **0.0076** | **0.60704** |
|  | P-GCA+M-CTC-48 | 0.04393 | 0.48055 |  | P-GTT+M-CTT-34 | 0.02655 | 0.49542 |
|  | P-GCA+M-CTC-55 | 0.04393 | 0.50055 |  | P-CCA+M-CTT-32 | 0.02823 | 0.45487 |
|  | E-AGG+M-CGT-37 | 0.04596 | 0.47017 |  | **E-AGG+M-CGT-22** | **0.00463** | **0.56304** |
|  | P-GTT+M-ATC-40 | 0.01975 | 0.42679 |  | P-CCA+M-CTT-7 | 0.03849 | 0.4921 |
|  | E-AAC+M-CTT-21 | 0.01279 | 0.42321 |  | P-TGG+M-TTG-54 | 0.03982 | 0.4918 |
|  | E-ACC+M-CAG-42 | 0.01185 | 0.49122 |  | P-TGG+M-TTG-55 | 0.03982 | 0.4918 |
| STL | E-ACC+M-CAG-35 | 0.00304 | 0.49438 |  | P-TGG+M-TTG-60 | 0.03982 | 0.4918 |
|  | P-GTT+M-CTT-22 | 0.01073 | 0.50412 |  | P-TGG+M-TTG-61 | 0.03982 | 0.4918 |
|  | E-AGA+M-CAG-12 | 0.00501 | 0.47988 |  | P-GTT+M-CTT-16 | 0.04329 | 0.49106 |
|  | E-AGA+M-CAG-15 | 0.00623 | 0.48755 |  | **P-TGG+M-CTT-33** | **0.00408** | **0.53009** |
|  | E-AAG+M-TGT-58 | 0.00707 | 0.45643 |  | P-CCA+M-CTT-9 | 0.04839 | 0.49008 |
|  | **P-CCA+M-AGA-49** | **0.00155** | **0.75213** | NLP | **P-CCA+M-AGA-49** | **3.04E-04** | **0.5408** |
|  | E-AAC+M-CCA-55 | 0.01527 | 0.48026 |  | E-AAC+M-TGT-3 | 0.006 | 0.51038 |
|  | P-CCA+M-CTT-9 | 0.022 | 0.50647 |  | P-TGG+M-CTT-29 | 0.01618 | 0.50079 |
|  | P-ACC+M-AGT-26 | 0.02361 | 0.44586 |  | P-TGG+M-CTT-18 | 0.01971 | 0.49892 |
|  | P-TGG+M-TTG-53 | 0.0271 | 0.47467 |  | P-CCA+M-AGA-50 | 0.01971 | 0.49892 |
|  | P-TGG+M-TTG-54 | 0.02793 | 0.46441 |  | P-CCA+M-CTT-7 | 0.02691 | 0.49601 |
|  | P-TGG+M-TTG-60 | 0.02793 | 0.48441 |  | P-CCA+M-CTT-32 | 0.03337 | 0.49402 |
|  | P-TGG+M-TTG-61 | 0.03283 | 0.50303 |  | E-AGG+M-CGT-37 | 0.03706 | 0.49306 |
|  | P-GTT+M-ATC-39 | 0.03305 | 0.42297 |  | **E-AGG+M-CGT-22** | **0.00759** | **0.61293** |
|  | P-ACC+M-AGA-46 | 0.03705 | 0.45199 |  | E-AAC+M-CTT-34 | 0.03886 | 0.49263 |
|  | E-AGG+M-CGT-37 | 0.03412 | 0.47289 |  | P-TGG+M-CTT-36 | 0.04126 | 0.49208 |
|  | P-TGG+M-TTG-53 | 0.03236 | 0.47618 |  | P-TGG+M-CTT-61 | 0.04364 | 0.49158 |
|  | E-ACC+M-CAG-12 | 0.03172 | 0.47242 |  | P-TGG+M-TTG-64 | 0.04513 | 0.49127 |
| NLP | P-ACC+M-AGA-46 | 4.28E-04 | 0.51148 |  | P-TGG+M-CTT-37 | 0.04658 | 0.49099 |
|  | P-GTT+M-ATC-39 | 4.28E-04 | 0.51148 | LL | P-GCA+M-AGT-24 | 0.01151 | 0.51231 |
|  | **P-CCA+M-AGA-49** | **5.81E-04** | **0.61874** |  | P-GCA+M-AGT-33 | 0.01311 | 0.51075 |
|  | E-AAC+M-CCA-55 | 0.0015 | 0.50023 |  | P-GCA+M-AGT-5 | 0.01379 | 0.51015 |
|  | E-AAG+M-TGT-58 | 0.0019 | 0.49813 |  | P-GCA+M-CTC-39 | 0.0147 | 0.50939 |
|  | E-ACC+M-CAG-36 | 0.0019 | 0.49813 |  | P-GCA+M-CTC-4 | 0.01919 | 0.50625 |
|  | E-ACC+M-CAG-39 | 0.00193 | 0.49846 |  | P-GCA+M-CTC-5 | 0.01919 | 0.50625 |
|  | E-AAC+M-TGT-29 | 0.00195 | 0.49792 |  | P-GCA+M-CTC-6 | 0.01919 | 0.50625 |
|  | E-AAC+M-TGT-3 | 0.00404 | 0.4914 |  | P-TGG+M-CTT-5 | 0.02686 | 0.50234 |
|  | E-AGG+M-CGT-37 | 0.01013 | 0.48326 |  | **P-TGG+M-CTT-33** | **0.00512** | **0.59927** |
|  | P-TGG+M-CTT-18 | 0.01267 | 0.48129 |  | P-GCA+M-CTC-40 | 0.03664 | 0.49878 |
|  | P-TGG+M-CTT-29 | 0.01288 | 0.48114 |  | E-AAC+M-TGT-24 | 0.04247 | 0.49711 |
|  | P-CCA+M-AGA-50 | 0.01288 | 0.48114 |  | **P-CCA+M-AGA-49** | **0.04466** | **0.55655** |
|  | E-AGA+M-CAG-20 | 0.01749 | 0.47847 | LW | P-TGG+M-CTT-37 | 0.00277 | 0.51613 |
|  | P-TGG+M-CTT-35 | 0.02028 | 0.47718 |  | **P-TGG+M-CTT-33** | **0.00534** | **0.54004** |
|  | E-AGA+M-CAG-9 | 0.02028 | 0.47718 |  | P-TGG+M-CTT-36 | 0.01783 | 0.49867 |
|  | E-AGA+M-CAG-15 | 0.02028 | 0.47718 | IL | P-CCA+M-CTT-32 | 0.01408 | 0.50124 |
|  | E-AGC+M-CGA-36 | 0.02286 | 0.47614 |  | P-TGG+M-CTT-37 | 0.01418 | 0.50118 |
|  | E-AGC+M-CTT-19 | 0.02434 | 0.4756 |  | P-TGG+M-TTG-16 | 0.01718 | 0.4994 |
|  | E-ACC+M-CAG-42 | 0.02559 | 0.47543 |  | P-TGG+M-TTG-10 | 0.01852 | 0.49871 |
|  | E-ACC+M-CAG-35 | 0.0262 | 0.47496 |  | **P-CCA+M-AGA-49** | **0.02021** | **0.59791** |
|  | E-AAC+M-CCA-7 | 0.03477 | 0.47254 |  | P-TGG+M-CTT-13 | 0.02339 | 0.49657 |
|  | P-TGG+M-TTG-53 | 0.03681 | 0.45281 |  | **E-AGG+M-CGT-22** | **0.03075** | **0.65441** |
| LL | E-AAG+M-TGT-58 | 6.09E-05 | 0.52894 |  | P-CCA+M-CTT-34 | 0.03109 | 0.494 |
|  | E-ACC+M-CAG-36 | 6.09E-05 | 0.52894 |  | P-TGG+M-TTG-18 | 0.03376 | 0.49327 |
|  | E-ACC+M-CAG-39 | 6.61E-05 | 0.52897 |  | E-AGG+M-CGT-16 | 0.036 | 0.49269 |
|  | E-ACC+M-CAG-35 | 4.00E-04 | 0.51271 | NF | **E-AGG+M-CGT-22** | 0.00477 | 0.61177 |
|  | E-ACC+M-CAG-42 | 4.90E-04 | 0.51027 |  | P-TGG+M-CTT-37 | 0.0145 | 0.50119 |
|  | E-AAC+M-CCA-55 | 0.00119 | 0.50229 |  | P-TGG+M-CTT-13 | 0.01532 | 0.50067 |
|  | E-AAG+M-TGT-49 | 0.00171 | 0.49908 |  | E-AGG+M-CGT-37 | 0.01835 | 0.499 |
|  | E-AAG+M-TGT-59 | 0.00171 | 0.49908 |  | **P-CCA+M-AGA-49** | **0.01926** | **0.96855** |
|  | E-AAG+M-TGT-60 | 0.00171 | 0.49908 |  | **P-TGG+M-CTT-33** | **0.0039** | **0.61656** |
|  | E-AAG+M-TGT-64 | 0.00171 | 0.49908 |  | P-GTT+M-CTT-17 | 0.02407 | 0.4965 |
|  | E-AAG+M-TGT-59 | 0.00171 | 0.49908 |  | E-AGG+M-CGT-41 | 0.04496 | 0.49087 |
|  | E-ACA+M-AAG-19 | 0.00171 | 0.49908 |  | P-GTT+M-CTT-4 | 0.04642 | 0.49059 |
|  | E-ACA+M-AAG-22 | 0.00171 | 0.49908 |  | P-TGG+M-CTT-36 | 0.04758 | 0.49037 |
|  | E-ACA+M-AAG-28 | 0.00171 | 0.49908 |  | **E-AGG+M-CGT-22** | **0.00477** | **1.11177** |
|  | E-ACA+M-AAG-34 | 0.00171 | 0.49908 | LET | E-AGG+M-CGT-34 | 0.00996 | 0.50664 |
|  | E-ACC+M-CAG-17 | 0.00171 | 0.49908 |  | E-AGG+M-CGT-41 | 0.011 | 0.50565 |
|  | E-ACC+M-CAG-25 | 0.00171 | 0.49908 |  | E-AGG+M-CGT-39 | 0.01117 | 0.5055 |
|  | E-ACC+M-CAG-32 | 0.00172 | 0.50186 |  | **P-TGG+M-CTT-33** | **0.01429** | **0.64305** |
|  | E-ACA+M-AAG-8 | 0.00175 | 0.49909 |  | E-AGG+M-CGT-45 | 0.02254 | 0.4986 |
|  | E-AGG+M-CGT-47 | 0.00175 | 0.49909 |  | P-GTT+M-CTT-34 | 0.02645 | 0.49705 |
|  | P-TGG+M-CTT-5 | 0.00631 | 0.48743 |  | P-GCA+M-AGT-33 | 0.03973 | 0.49318 |
|  | **P-CCA+M-AGA-49** | **0.0073** | **0.87614** | WET | E-AAC+M-CCA-7 | 0.00687 | 0.50918 |
|  | P-GCA+M-AGT-5 | 0.01277 | 0.48122 |  | P-CCA+M-CTT-48 | 0.01164 | 0.50404 |
|  | E-ACC+M-CAG-12 | 0.01332 | 0.48085 |  | E-AAC+M-CTT-21 | 0.01544 | 0.50133 |
|  | E-ACC+M-CAG-20 | 0.01332 | 0.48085 |  | P-GTT+M-CTT-18 | 0.01762 | 0.50007 |
|  | E-ACC+M-CAG-21 | 0.01332 | 0.48085 |  | P-TGG+M-TTG-53 | 0.01907 | 0.49932 |
|  | E-ACC+M-CAG-30 | 0.01332 | 0.48085 | LIT | **P-TGG+M-CTT-33** | **0.0127** | **0.57257** |
|  | E-ACC+M-CAG-31 | 0.01332 | 0.48085 |  | P-GCA+M-CTC-39 | 0.0145 | 0.50132 |
|  | E-AGA+M-CAG-9 | 0.01409 | 0.48083 |  | P-TGG+M-CTT-13 | 0.02471 | 0.49637 |
|  | P-ACC+M-AGA-46 | 0.01436 | 0.48019 |  | E-AAC+M-CTT-2 | 0.02705 | 0.49555 |
| LW | P-TGG+M-CTT-5 | 5.72E-04 | 0.51007 |  | E-AAC+M-CTT-4 | 0.02705 | 0.49555 |
|  | P-TGG+M-CTT-28 | 0.00117 | 0.50246 |  | E-AAC+M-TGT-45 | 0.0412 | 0.49174 |
|  | **P-TGG+M-CTT-33** | **0.00446** | **1.03052** |  | E-AAC+M-TGT-51 | 0.0424 | 0.49149 |
|  | P-TGG+M-CTT-36 | 0.00637 | 0.48891 |  | P-TGG+M-CTT-43 | 0.0431 | 0.49134 |
|  | P-TGG+M-CTT-37 | 0.0146 | 0.48004 |  | P-TGG+M-CTT-28 | 0.04653 | 0.49066 |
|  | P-TGG+M-TTG-64 | 0.02818 | 0.47471 | WIT | E-AAG+M-TGT-9 | 0.00375 | 0.51574 |
|  | P-GTT+M-CTT-21 | 0.03353 | 0.50285 |  | E-AAG+M-TGT-13 | 0.00375 | 0.51574 |
|  | P-GTT+M-CTT-26 | 0.03569 | 0.43231 |  | E-AAG+M-TGT-20 | 0.00375 | 0.51574 |
|  | P-GTT+M-CTT-28 | 0.03743 | 0.50191 |  | P-GTT+M-CTT-38 | 0.00818 | 0.50793 |
|  | P-GTT+M-ATC-50 | 0.04041 | 0.47215 |  | E-AAC+M-CTT-2 | 0.01187 | 0.50427 |
|  | P-ACC+M-AGT-26 | 0.04318 | 0.4707 |  | E-AAC+M-CTT-4 | 0.01187 | 0.50427 |
|  | E-AAC+M-CTT-5 | 0.04889 | 0.46965 |  | P-CCA+M-CTT-51 | 0.02315 | 0.49784 |
| IL | **P-TGG+M-CTT-33** | **0.00823** | **0.92509** |  | P-TGG+M-TTG-76 | 0.03263 | 0.4946 |
|  | P-TGG+M-CTT-37 | 0.0137 | 0.4806 |  | P-ACC+M-AGT-27 | 0.04831 | 0.49098 |
|  | P-TGG+M-CTT-70 | 0.0218 | 0.47655 | LLI | E-AGC+M-CGA-21 | 0.0015 | 0.5233 |
|  | P-TGG+M-CTT-72 | 0.0325 | 0.47311 |  | E-AAC+M-CCA-32 | 0.0057 | 0.51015 |
|  | P-TGG+M-TTG-1 | 0.03371 | 0.48315 |  | P-GTT+M-CTT-34 | 0.01252 | 0.50264 |
|  | P-TGG+M-TTG-16 | 0.03854 | 0.47166 |  | P-GTT+M-CTT-18 | 0.0145 | 0.50126 |
|  | **P-CCA+M-AGA-49** | **0.0004** | **0.84126** |  | E-AGG+M-CGT-35 | 0.01534 | 0.50073 |
|  | P-ACC+M-AGA-66 | 0.04083 | 0.43117 |  | E-AAC+M-CTT-21 | 0.01618 | 0.50023 |
|  | P-CCA+M-CTT-32 | 0.04234 | 0.50086 |  | **E-AGG+M-CGT-22** | **0.00285** | **0.59704** |
|  | **E-AGG+M-CGT-22** | **0.00315** | **0.5707** |  | P-TGG+M-CTT-13 | 0.03476 | 0.45322 |
|  | E-AGA+M-CAG-12 | 0.00501 | 0.48988 |  | **P-CCA+M-AGA-49** | **0.00553** | **0.54302** |
|  | E-ACC+M-CAG-42 | 0.00409 | 0.4913 |  | P-CCA+M-CTT-9 | 0.03747 | 0.49255 |
|  | E-ACC+M-CAG-36 | 0.00469 | 0.49007 |  | P-TGG+M-CTT-36 | 0.0393 | 0.49212 |
|  | E-ACC+M-CAG-39 | 0.00469 | 0.49007 |  | E-AGC+M-CTT-6 | 0.04501 | 0.49091 |
|  | E-AGG+M-CGT-37 | 0.04534 | 0.47028 |  | E-AAG+M-TGT-47 | 0.04955 | 0.49006 |
| NF | E-AGG+M-CGT-37 | 0.00309 | 0.49379 | WL | P-GTT+M-CTT-34 | 0.01112 | 0.50334 |
|  | **E-AGG+M-CGT-22** | **0.00429** | **0.79087** |  | E-AGC+M-CGA-21 | 0.01625 | 0.49982 |
|  | E-AGG+M-CGT-41 | 0.00997 | 0.48339 |  | E-AGA+M-CAG-43 | 0.01983 | 0.49799 |
|  | **P-TGG+M-CTT-33** | **0.0026** | **1.01133** |  | P-GTT+M-ATC-22 | 0.0269 | 0.49523 |
|  | P-GTT+M-CTT-26 | 0.01602 | 0.48046 |  | E-AGG+M-CGT-35 | 0.0327 | 0.49348 |
|  | P-TGG+M-CTT-37 | 0.01651 | 0.47897 |  | **E-AGG+M-CGT-22** | **0.001** | **0.57287** |
|  | **P-CCA+M-AGA-49** | **0.00666** | **0.92889** |  | P-TGG+M-CTT-36 | 0.03874 | 0.49197 |
|  | P-GTT+M-CTT-21 | 0.0256 | 0.47622 |  | P-TGG+M-CTT-76 | 0.04941 | 0.48984 |
|  | E-AAC+M-CCA-7 | 0.02964 | 0.4739 | LMLL | E-AAC+M-CTT-21 | 0.00865 | 0.50561 |
|  | E-AAC+M-CCA-6 | 0.03939 | 0.47192 |  | P-TGG+M-TTG-2 | 0.01149 | 0.50295 |
|  | E-AAG+M-TGT-58 | 0.04294 | 0.47074 |  | **P-CCA+M-AGA-49** | **0.00403** | **0.5511** |
|  | E-ACC+M-CAG-36 | 0.04294 | 0.47074 |  | P-CCA+M-CTT-32 | 0.02267 | 0.49671 |
|  | P-TGG+M-CTT-36 | 0.04344 | 0.47064 |  | P-TGG+M-TTG-25 | 0.02991 | 0.49421 |
|  | E-ACC+M-CAG-39 | 0.04397 | 0.47075 |  | E-AGG+M-CGT-35 | 0.03819 | 0.49204 |
|  | E-AGG+M-CGT-33 | 0.04594 | 0.47017 |  | P-GTT+M-CTT-34 | 0.04144 | 0.49133 |
|  | E-AAC+M-CCA-55 | 0.04741 | 0.46991 |  | P-TGG+M-CTT-13 | 0.04988 | 0.48971 |
|  | P-CCA+M-CTT-55 | 0.04749 | 0.46989 | LMLW | E-AAC+M-CCA-53 | 0.01143 | 0.50342 |
|  | P-CCA+M-CTT-57 | 0.04749 | 0.46989 |  | E-AAC+M-CTT-21 | 0.01952 | 0.49842 |
| **LET** | **P-TGG+M-CTT-33** | **0.00626** | **0.63751** |  | P-GTT+M-CTT-18 | 0.02042 | 0.49801 |
|  | P-GTT+M-CTT-26 | 0.01166 | 0.48336 |  | P-TGG+M-CTT-5 | 0.04249 | 0.49137 |
|  | P-GTT+M-CTT-21 | 0.01187 | 0.4832 |  | E-AAC+M-TGT-3 | 0.04739 | 0.49041 |
|  | P-GTT+M-CTT-28 | 0.01282 | 0.48166 |  | P-TGG+M-TTG-43 | 0.04873 | 0.49016 |
|  | P-GTT+M-CTT-34 | 0.01307 | 0.48101 | LLL | E-AGA+M-CAG-43 | 0.01336 | 0.50201 |
|  | P-GCA+M-AGT-33 | 0.02318 | 0.47602 |  | P-TGG+M-TTG-25 | 0.01833 | 0.49906 |
|  | E-AAC+M-TGT-35 | 0.03295 | 0.473 |  | P-TGG+M-CTT-37 | 0.0198 | 0.49834 |
|  | P-GCA+M-CTC-39 | 0.03618 | 0.4722 |  | E-AGC+M-CGA-11 | 0.0391 | 0.49215 |
|  | P-CCA+M-CTT-39 | 0.04685 | 0.47 |  | P-TGG+M-CTT-13 | 0.04753 | 0.49042 |
| WET | P-CCA+M-CTT-48 | 0.00444 | 0.49057 | SW | P-TGG+M-TTG-25 | 0.00836 | 0.50844 |
|  | E-AAC+M-CTT-18 | 0.00827 | 0.48505 |  | P-TGG+M-CTT-36 | 0.0232 | 0.49835 |
|  | P-TGG+M-TTG-53 | 0.00897 | 0.48432 |  | E-AAC+M-CTT-21 | 0.03218 | 0.49521 |
|  | E-AAC+M-CCA-6 | 0.0093 | 0.4847 |  | P-TGG+M-TTG-64 | 0.0432 | 0.49242 |
|  | E-AAC+M-CCA-7 | 0.00962 | 0.48371 | BFW | P-TGG+M-CTT-58 | 0.00143 | 0.52586 |
|  | P-GTT+M-CTT-18 | 0.01381 | 0.48053 |  | P-TGG+M-CTT-66 | 0.00559 | 0.51189 |
|  | E-ACC+M-CAG-39 | 0.01391 | 0.48078 |  | **P-CCA+M-AGA-49** | **0.00718** | **0.61082** |
|  | E-AAG+M-TGT-58 | 0.01435 | 0.4802 |  | P-CCA+M-CTT-51 | 0.03504 | 0.49404 |
|  | E-ACC+M-CAG-36 | 0.01422 | 0.4801 |  | E-AAC+M-CCA-49 | 0.03994 | 0.49282 |
|  | E-AAC+M-CCA-55 | 0.01456 | 0.48007 |  | E-AAC+M-CCA-52 | 0.03994 | 0.49282 |
|  | P-ACC+M-AGA-46 | 0.02644 | 0.47489 |  | P-ACC+M-AGA-52 | 0.04912 | 0.49091 |
|  | E-ACC+M-CAG-42 | 0.03358 | 0.47307 | BDW | P-TGG+M-CTT-58 | 0.00201 | 0.52281 |
|  | E-AGC+M-CGA-19 | 0.03373 | 0.4728 |  | P-TGG+M-CTT-66 | 0.0094 | 0.50708 |
|  | E-ACC+M-CAG-35 | 0.03597 | 0.47225 |  | **P-CCA+M-AGA-49** | **0.02284** | **0.66837** |
|  | P-CCA+M-CTT-32 | 0.03727 | 0.47194 |  | P-CCA+M-CTT-51 | 0.02517 | 0.49743 |
|  | P-GTT+M-CTT-26 | 0.04341 | 0.47152 |  | E-AAC+M-CCA-49 | 0.03885 | 0.49331 |
|  | P-CCA+M-CTT-25 | 0.04491 | 0.47036 |  | E-AAC+M-CCA-52 | 0.03885 | 0.49331 |
|  | P-CCA+M-CTT-29 | 0.04491 | 0.47036 | GLN | E-AAG+M-TGT-47 | 0.00395 | 0.51358 |
| **LIT** | **P-TGG+M-CTT-33** | **0.01026** | **0.65314** |  | **E-AGG+M-CGT-22** | **0.00292** | **0.58892** |
|  | E-AAC+M-CTT-2 | 0.0193 | 0.47761 |  | E-AAC+M-TGT-3 | 0.02614 | 0.49572 |
|  | E-AAC+M-CTT-4 | 0.0193 | 0.47761 |  | E-AAC+M-CCA-32 | 0.02794 | 0.49511 |
|  | P-GCA+M-CTC-39 | 0.02282 | 0.47616 |  | E-AGA+M-CAG-43 | 0.03296 | 0.49362 |
|  | E-AAG+M-TGT-58 | 0.0274 | 0.47458 |  | **P-TGG+M-CTT-33** | **0.00562** | **0.56292** |
|  | E-ACC+M-CAG-36 | 0.0274 | 0.47458 |  | E-AGA+M-CAG-30 | 0.03978 | 0.49194 |
|  | E-ACC+M-CAG-39 | 0.0287 | 0.47443 | SCH | E-AGG+M-CGT-35 | 0.03424 | 0.49506 |
|  | E-AAG+M-TGT-4 | 0.04133 | 0.47107 |  | E-AAC+M-TGT-29 | 0.03642 | 0.47446 |
|  | E-AAC+M-TGT-45 | 0.04206 | 0.47092 |  | E-AGG+M-CGT-37 | 0.045 | 0.49243 |
|  | P-TGG+M-CTT-13 | 0.04254 | 0.47082 |  | E-AGC+M-CGA-21 | 0.0476 | 0.4919 |
|  | P-TGG+M-CTT-43 | 0.04574 | 0.47021 | ATC | E-AAC+M-CCA-32 | 0.0228 | 0.49694 |
| WIT | E-AAG+M-TGT-9 | 0.004 | 0.49148 |  | P-TGG+M-TTG-25 | 0.02853 | 0.4949 |
|  | E-AAG+M-TGT-13 | 0.004 | 0.49148 |  | E-AAC+M-CTT-21 | 0.02876 | 0.49482 |
|  | E-AAG+M-TGT-20 | 0.004 | 0.49148 |  | E-AGA+M-CAG-43 | 0.03549 | 0.49293 |
|  | P-GTT+M-CTT-38 | 0.00822 | 0.4851 |  | E-AAC+M-TGT-29 | 0.04154 | 0.49153 |
|  | E-AAC+M-CTT-2 | 0.00836 | 0.48495 |  | **E-AGG+M-CGT-22** | **0.00174** | **0.59149** |
|  | E-AAC+M-CTT-4 | 0.00836 | 0.48495 |  | E-AAG+M-TGT-47 | 0.04832 | 0.49019 |
|  | E-AAC+M-CCA-50 | 0.02395 | 0.47574 |  |  |  |  |
|  | E-AAC+M-CCA-53 | 0.02395 | 0.47574 |  |  |  |  |
|  | P-TGG+M-TTG-76 | 0.02424 | 0.47564 |  |  |  |  |
|  | P-TGG+M-CTT-18 | 0.04156 | 0.47102 |  |  |  |  |
|  | P-CCA+M-AGA-50 | 0.04156 | 0.47102 |  |  |  |  |
|  | P-CCA+M-CTT-58 | 0.04417 | 0.4705 |  |  |  |  |
|  | P-ACC+M-AGT-27 | 0.0455 | 0.47025 |  |  |  |  |
|  | P-CCA+M-CTT-51 | 0.04978 | 0.46949 |  |  |  |  |
| LLI | P-GTT+M-CTT-34 | 0.00451 | 0.49043 |  |  |  |  |
|  | E-AGC+M-CGA-32 | 0.01618 | 0.47915 |  |  |  |  |
|  | P-ACC+M-AGT-26 | 0.01838 | 0.47861 |  |  |  |  |
|  | E-AGC+M-CGA-36 | 0.02275 | 0.47619 |  |  |  |  |
|  | E-AGC+M-CGA-17 | 0.02295 | 0.47611 |  |  |  |  |
|  | **P-CCA+M-AGA-49** | **0.02706** | **0.57469** |  |  |  |  |
|  | P-TGG+M-CTT-36 | 0.03516 | 0.47244 |  |  |  |  |
|  | P-CCA+M-CTT-9 | 0.03602 | 0.47223 |  |  |  |  |
|  | **P-TGG+M-CTT-33** | **0.04527** | **0.57029** |  |  |  |  |
|  | E-AGC+M-CGA-30 | 0.04543 | 0.47027 |  |  |  |  |
|  | P-CCA+M-CTT-55 | 0.04651 | 0.47007 |  |  |  |  |
|  | P-CCA+M-CTT-57 | 0.04651 | 0.47007 |  |  |  |  |
|  | P-GTT+M-CTT-26 | 0.04669 | 0.47088 |  |  |  |  |
|  | E-AAG+M-TGT-58 | 0.0469 | 0.47 |  |  |  |  |
|  | E-ACC+M-CAG-36 | 0.0469 | 0.47 |  |  |  |  |
|  | E-ACC+M-CAG-39 | 0.04787 | 0.47003 |  |  |  |  |
|  | E-AAC+M-CCA-23 | 0.04791 | 0.46992 |  |  |  |  |
|  | E-AAC+M-CCA-42 | 0.04986 | 0.46948 |  |  |  |  |
| WL | P-ACC+M-AGT-26 | 6.83E-04 | 0.50846 |  |  |  |  |
|  | P-GTT+M-CTT-26 | 0.0122 | 0.48294 |  |  |  |  |
|  | P-GTT+M-CTT-34 | 0.01761 | 0.45841 |  |  |  |  |
|  | **E-AGG+M-CGT-22** | **0.00666** | **0.97482** |  |  |  |  |
|  | E-AGG+M-CGT-37 | 0.03012 | 0.47377 |  |  |  |  |
|  | P-GTT+M-ATC-22 | 0.03779 | 0.49183 |  |  |  |  |
|  | E-AGG+M-CGT-35 | 0.04506 | 0.47033 |  |  |  |  |
| LMLL | P-GTT+M-CTT-26 | 0.00643 | 0.48883 |  |  |  |  |
|  | P-TGG+M-TTG-2 | 0.00698 | 0.42654 |  |  |  |  |
|  | P-GTT+M-CTT-28 | 0.00774 | 0.48617 |  |  |  |  |
|  | **P-TGG+M-CTT-33** | **0.0088** | **0.71449** |  |  |  |  |
|  | **P-CCA+M-AGA-49** | **0.00927** | **0.55404** |  |  |  |  |
|  | E-ACC+M-CAG-42 | 0.01058 | 0.43321 |  |  |  |  |
|  | E-ACC+M-CAG-35 | 0.01197 | 0.48179 |  |  |  |  |
|  | P-GTT+M-CTT-34 | 0.01593 | 0.47928 |  |  |  |  |
|  | **E-AGG+M-CGT-22** | **0.00643** | **0.54901** |  |  |  |  |
|  | E-AAG+M-TGT-58 | 0.02229 | 0.47636 |  |  |  |  |
|  | E-ACC+M-CAG-36 | 0.02229 | 0.47636 |  |  |  |  |
|  | E-AGG+M-CGT-35 | 0.02284 | 0.47615 |  |  |  |  |
|  | E-ACC+M-CAG-39 | 0.02289 | 0.5164 |  |  |  |  |
|  | E-AGA+M-CAG-12 | 0.02781 | 0.50445 |  |  |  |  |
|  | E-AGA+M-CAG-15 | 0.02781 | 0.47445 |  |  |  |  |
|  | E-AAC+M-CCA-55 | 0.02784 | 0.47444 |  |  |  |  |
|  | E-AGG+M-CGT-37 | 0.02928 | 0.47401 |  |  |  |  |
|  | P-TGG+M-CTT-65 | 0.03549 | 0.47236 |  |  |  |  |
|  | P-ACC+M-AGT-26 | 0.04025 | 0.47173 |  |  |  |  |
|  | P-TGG+M-CTT-36 | 0.04505 | 0.47034 |  |  |  |  |
| LMLW | E-AAG+M-TGT-18 | 0.00765 | 0.48573 |  |  |  |  |
|  | E-AGA+M-CAG-48 | 0.01893 | 0.47778 |  |  |  |  |
|  | E-AGA+M-CAG-49 | 0.01893 | 0.47778 |  |  |  |  |
|  | P-TGG+M-CTT-5 | 0.019 | 0.47775 |  |  |  |  |
|  | **P-TGG+M-CTT-33** | **0.02545** | **0.59422** |  |  |  |  |
|  | P-GTT+M-CTT-34 | 0.02801 | 0.47439 |  |  |  |  |
|  | P-TGG+M-TTG-2 | 0.03072 | 0.4736 |  |  |  |  |
|  | P-GTT+M-CTT-26 | 0.03563 | 0.47327 |  |  |  |  |
|  | P-CCA+M-AGA-28 | 0.04964 | 0.46952 |  |  |  |  |
| LLL | E-AAG+M-TGT-58 | 0.00118 | 0.5024 |  |  |  |  |
|  | E-ACC+M-CAG-36 | 0.00118 | 0.5024 |  |  |  |  |
|  | E-ACC+M-CAG-39 | 0.00125 | 0.50239 |  |  |  |  |
|  | E-ACC+M-CAG-42 | 0.00125 | 0.50241 |  |  |  |  |
|  | E-ACC+M-CAG-35 | 0.00146 | 0.50046 |  |  |  |  |
|  | E-AAG+M-TGT-49 | 0.00165 | 0.49937 |  |  |  |  |
|  | E-AAG+M-TGT-59 | 0.00165 | 0.49937 |  |  |  |  |
|  | E-AAG+M-TGT-60 | 0.00165 | 0.49937 |  |  |  |  |
|  | E-AAG+M-TGT-64 | 0.00165 | 0.49937 |  |  |  |  |
|  | E-AAC+M-CCA-55 | 0.01021 | 0.48319 |  |  |  |  |
|  | E-AAG+M-TGT-27 | 0.01476 | 0.47995 |  |  |  |  |
|  | E-AAG+M-TGT-34 | 0.01476 | 0.47995 |  |  |  |  |
|  | E-ACA+M-AAG-10 | 0.01476 | 0.47995 |  |  |  |  |
|  | E-ACA+M-AAG-24 | 0.01476 | 0.47995 |  |  |  |  |
|  | E-ACC+M-CAG-13 | 0.01476 | 0.47995 |  |  |  |  |
|  | E-ACC+M-CAG-14 | 0.01476 | 0.47995 |  |  |  |  |
|  | E-ACC+M-CAG-49 | 0.01476 | 0.47995 |  |  |  |  |
|  | E-ACC+M-CAG-50 | 0.01476 | 0.47995 |  |  |  |  |
|  | E-ACA+M-AAG-42 | 0.01499 | 0.48012 |  |  |  |  |
|  | P-TGG+M-CTT-37 | 0.01712 | 0.47866 |  |  |  |  |
|  | P-GTT+M-CTT-26 | 0.02865 | 0.47521 |  |  |  |  |
|  | E-AGA+M-CAG-12 | 0.03686 | 0.47204 |  |  |  |  |
|  | E-AGA+M-CAG-15 | 0.03686 | 0.47204 |  |  |  |  |
|  | P-TGG+M-CTT-36 | 0.03862 | 0.47164 |  |  |  |  |
|  | E-AAC+M-CCA-42 | 0.04614 | 0.47013 |  |  |  |  |
| SW | P-ACC+M-AGT-26 | 0.00106 | 0.50441 |  |  |  |  |
|  | P-TGG+M-CTT-36 | 0.02025 | 0.4772 |  |  |  |  |
|  | P-CCA+M-CTT-51 | 0.03316 | 0.47294 |  |  |  |  |
|  | P-TGG+M-TTG-64 | 0.0414 | 0.47105 |  |  |  |  |
| BFW | P-TGG+M-CTT-58 | 9.85E-04 | 0.50401 |  |  |  |  |
|  | P-CCA+M-AGA-49 | 0.00864 | 0.48465 |  |  |  |  |
|  | P-ACC+M-AGA-53 | 0.01418 | 0.48046 |  |  |  |  |
|  | P-GCA+M-CTC-15 | 0.01916 | 0.47767 |  |  |  |  |
|  | P-CCA+M-CTT-51 | 0.02097 | 0.47689 |  |  |  |  |
|  | P-ACC+M-AGA-52 | 0.02296 | 0.47611 |  |  |  |  |
|  | E-AAC+M-TGT-35 | 0.02326 | 0.47599 |  |  |  |  |
|  | P-TGG+M-CTT-66 | 0.02348 | 0.47591 |  |  |  |  |
|  | E-AAC+M-CCA-49 | 0.0342 | 0.47268 |  |  |  |  |
|  | E-AAC+M-CCA-52 | 0.0342 | 0.47268 |  |  |  |  |
|  | E-ACA+M-AAG-16 | 0.04327 | 0.47068 |  |  |  |  |
|  | P-ACC+M-AGA-46 | 0.04872 | 0.46968 |  |  |  |  |
|  | P-GTT+M-CTT-18 | 0.04872 | 0.46968 |  |  |  |  |
| BDW | P-TGG+M-CTT-58 | 0.0013 | 0.50155 |  |  |  |  |
|  | **P-CCA+M-AGA-49** | **0.00174** | **0.83195** |  |  |  |  |
|  | P-CCA+M-CTT-51 | 0.01694 | 0.47875 |  |  |  |  |
|  | P-GCA+M-CTC-15 | 0.02575 | 0.47511 |  |  |  |  |
|  | P-ACC+M-AGA-53 | 0.02615 | 0.47511 |  |  |  |  |
|  | P-TGG+M-CTT-66 | 0.02785 | 0.47444 |  |  |  |  |
|  | E-AAC+M-TGT-35 | 0.03771 | 0.47184 |  |  |  |  |
|  | P-ACC+M-AGA-52 | 0.03973 | 0.4714 |  |  |  |  |
|  | E-AAC+M-CCA-49 | 0.04328 | 0.47067 |  |  |  |  |
|  | E-AAC+M-CCA-52 | 0.04328 | 0.47067 |  |  |  |  |
| GLN | E-AAC+M-TGT-29 | 0.01399 | 0.48042 |  |  |  |  |
|  | P-CCA+M-AGA-17 | 0.02921 | 0.47403 |  |  |  |  |
|  | P-ACC+M-AGT-26 | 0.03242 | 0.47361 |  |  |  |  |
|  | E-ACC+M-CAG-42 | 0.04262 | 0.47102 |  |  |  |  |
|  | E-ACC+M-CAG-35 | 0.04759 | 0.46987 |  |  |  |  |
| SCH | P-ACC+M-AGT-26 | 0.01291 | 0.48177 |  |  |  |  |
|  | E-ACC+M-CAG-42 | 0.0151 | 0.48006 |  |  |  |  |
|  | E-ACC+M-CAG-35 | 0.01696 | 0.47874 |  |  |  |  |
|  | **E-AGG+M-CGT-22** | **0.00232** | **0.62316** |  |  |  |  |
|  | **P-TGG+M-CTT-33** | **0.00621** | **0.77219** |  |  |  |  |
|  | E-AAG+M-TGT-58 | 0.04115 | 0.4711 |  |  |  |  |
|  | E-ACC+M-CAG-36 | 0.04115 | 0.4711 |  |  |  |  |
|  | E-AAC+M-TGT-29 | 0.0421 | 0.47091 |  |  |  |  |
|  | E-ACC+M-CAG-39 | 0.04257 | 0.47103 |  |  |  |  |
| TAC | P-TGG+M-TTG-53 | 0.00803 | 0.4853 |  |  |  |  |
|  | E-AGG+M-CGT-37 | 0.0205 | 0.47709 |  |  |  |  |
|  | **E-AGG+M-CGT-22** | **0.00464** | **0.89455** |  |  |  |  |
|  | E-AGG+M-CGT-41 | 0.0343 | 0.47265 |  |  |  |  |
|  | **P-TGG+M-CTT-33** | **0.00861** | **0.95164** |  |  |  |  |
|  | E-AGG+M-CGT-1 | 0.03925 | 0.4715 |  |  |  |  |
|  | E-AGG+M-CGT-4 | 0.04628 | 0.47118 |  |  |  |  |
